# A survey among geriatricians on geriatric oncology and geriatric surgical oncology models in Europe

**DOI:** 10.1007/s41999-026-01575-y

**Published:** 2026-08-28

**Authors:** Paula Sobrini-Morillo, Isabelle Moens, Alfonso Cruz-Jentoft, Andrea Sebastiano Ciccone, Jugdeep K. Dhesi, Nicolas M. Gonzalez-Senac, Marije Hamaker, Cecilia Lund, Francesco Mattace-Raso, Nina Neuendorff, Siri Rostoft, David Shipway, Giuseppe Dario Testa, Anja Velghe, Harmke A. Polinder-Bos

**Affiliations:** 1 Department of Geriatric Medicine, Hospital Universitario Puerta de Hierro de Majadahonda, 1 Joaquín Rodrigo Street, Majadahonda, 28222 Madrid, Spain; 2 Department of Internal Medicine, Section Geriatric Medicine, Erasmus MC University Medical Center, Rotterdam, The Netherlands; 3 Servicio de Geriatría, Hospital Universitario Ramón y Cajal (IRYCIS), Madrid, Spain; 4 Department of Geriatric Medicine, Université Côte d’Azur, Sophia-Antipolis INSERM U1081, CNRS, UMR 7284, Centre Hospitalier Universitaire de Nice, Hôpital Pasteur 2, Nice, France; 5 Ageing and Health, Guy’s and St Thomas’ NHS Foundation Trust, London, UK; 6 Department of Geriatric Medicine, Hospital General Universitario Gregorio Marañón, Madrid, Spain; 7 Department of Geriatric Medicine, Diakonessenhuis, Utrecht, The Netherlands; 8 Department of Medicine, Copenhagen University Hospital, Herlev and Gentofte, Herlev, Denmark; 9 Department of Geriatric Medicine, Marien Hospital Herne, University Hospital of the Ruhr University Bochum, Herne, Germany; 10 Department of Geriatric Medicine, Oslo University Hospital, Oslo, Norway; 11 Department of Geriatric Medicine, Bristol NHS Group, Bristol, UK; 12 Division of Intensive and Cardiogeriatric Medicine, Careggi University Hospital, Florence, Italy; 13 Geriatric Department, Ghent University Hospital, Ghent, Belgium

**Keywords:** Geriatric oncology, Europe, Geriatric surgical oncology, Frailty

## Abstract

**Purpose:**

To provide an up-to-date overview of geriatricians’ involvement in oncological and onco-surgical care for older adults across Europe.

**Methods:**

An anonymous 35-item online survey was distributed via (inter)national societies between 10 April and 23 May 2025. The responses were summarized, and associations between comprehensive geriatric assessment (CGA) outputs, participation in oncological multidisciplinary team (MDT) meetings, and involvement in treatment decision-making were explored.

**Results:**

A total of 2160 responses were received from 37 countries. Further analysis included 395 geriatrician/internal medicine respondents with > 25% survey completion from 34 countries. Of them, 19% reported no formal geriatric oncology model; the most common approaches were referral based on clinical judgment (43%) and a two-step pathway (42%). Frailty screening-guided referral occurred in 63%, with the G8 (50%) and Clinical Frailty Scale (32%) being the most commonly used. Among respondents, 30% reported routine MDT attendance, and 20% reported involvement in treatment decisions. In exploratory analyses, routine MDT participation was associated with active treatment decision-making (aOR 6.01, 95% CI 3.08–11.7), CGA-based recommendations addressing both geriatric domains and oncological treatment (aOR 5.81, 95% CI 3.16–10.7), and positive feedback from oncology/surgery teams (aOR 3.75, 95% CI 1.96–7.14; all *p* < 0.001). Only 39% of respondents were involved in inpatient surgical oncology care, with liaison models predominating (37%).

**Conclusion:**

Geriatric oncology and geriatric surgical oncology remain unevenly implemented across Europe. Standardized pathways, training, and MDT integration may improve equity of care for older adults with cancer.

**Graphical abstract:**

**Supplementary Information:**

The online version contains supplementary material available at https://doi.org/10.1007/s41999-026-01575-y.

## Key summary points

**Aim:**

To describe and compare geriatricians’ roles in geriatric oncology and onco-surgical care across Europe.

**Findings:**

Geriatric oncology practices varied widely across 34 countries: 19% had no formal model, while main models were clinical-judgment referral (43%) and a two-step frailty screening plus comprehensive geriatric assessment (CGA) (42%). Oncological multidisciplinary team meeting (MDT) participation was associated with active treatment decision-making, broader CGA-based recommendations, and positive feedback from oncology/surgery teams. Geriatric surgical oncology appeared less developed, with only 39% of respondents involved in inpatient cancer surgery care. National education in geriatric (surgical) oncology and national oncological guidelines for older adults were often absent.

**Message:**

Standardized care pathways, geriatrician participation in the oncological MDTs, and stronger (inter)national training and guidelines are needed to improve equitable care for older patients with cancer in Europe.

**Supplementary Information:**

The online version contains supplementary material available at https://doi.org/10.1007/s41999-026-01575-y.

## Graphical abstract

**Figure:**
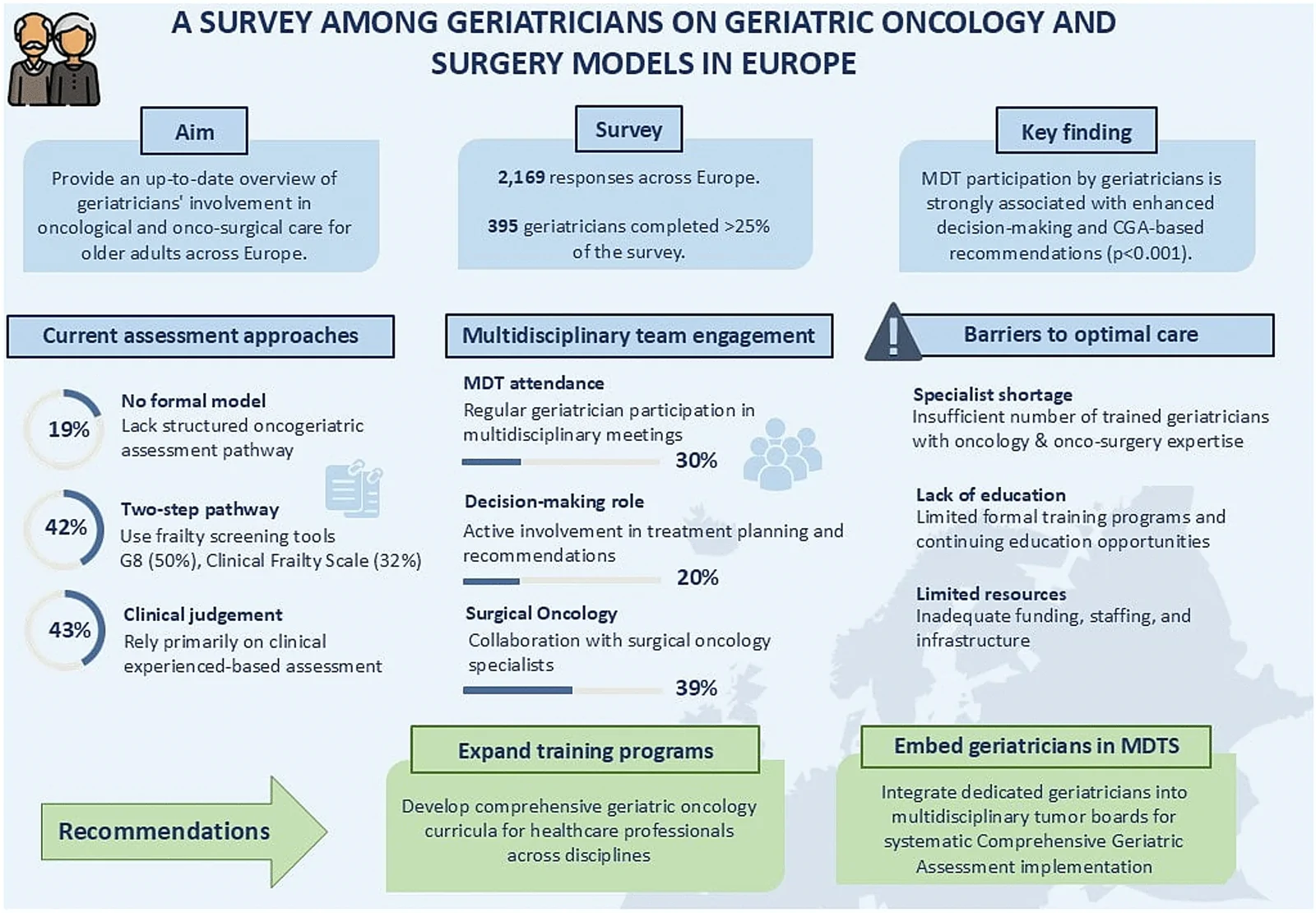

## Introduction

Older adults represent a growing proportion of people with cancer. This group includes both robust, active, and competent patients and malnourished and cognitively impaired patients living with frailty. Therefore, chronological age is an insufficient indicator of resilience, while frailty is more informative for selecting adequate treatment [1–3].

Current treatment guidelines and recommendations cannot simply be extrapolated to older patients, as such patients are often excluded from randomized controlled oncological and surgical trials [4]. However, novel, less intensive treatments (e.g., immunotherapy) and surgical innovations (e.g., minimally invasive surgery) are leading to treatment strategies that may be more accessible to older patients with cancer and frailty. To address this heterogeneity and help guiding treatment selection in older oncology patients, several scientific societies and organizations [5–9] recommend the use of a comprehensive geriatric assessment (CGA) or an abbreviated version, a geriatric assessment (GA), if CGA is unavailable.

CGA is a multidimensional evaluation performed by a geriatrician-led multidisciplinary team to identify medical, social, functional, and psychological needs, and to develop an integrated, coordinated care plan with interventions for all identified health issues [10]. Many of the tools and tests used in CGA can be incorporated into routine clinical practice and performed by non-geriatrician providers, without advanced training. This assessment, referred to as a GA, can be used to identify patients with high risk of poor outcomes and who may benefit from referral to a geriatrician [11, 12]. The integration of geriatric medicine in oncological care is known as ‘geriatric oncology’ or ‘oncogeriatrics’ [13]. It aims to implement interventions that improve diagnosis, facilitate appropriate treatment, prevent complications, and improve patient-reported outcomes [14].

The organization and implementation of cancer care for older patients, as well as access to it, differ between European countries [15, 16]. The lack of standardized approaches and the limited number of geriatricians in several countries contribute to large disparities and various care models in geriatric oncology across Europe [17]. In some countries, no formal geriatric oncology care is present. In others, where there are only a few geriatricians, a GA is performed by an oncologist or trained nurse, with no subsequent CGA. Where CGA-based models can be implemented, the two-step approach is commonly used [18, 19]. In this model, older patients are first screened for frailty, and those at risk are referred for a CGA to guide treatment and follow-up [5].

Principles of perioperative geriatric care, including early identification of frailty, CGA-based optimization, shared decision-making, and coordinated postoperative care, are directly relevant to older adults undergoing oncological surgery, in whom frailty, geriatric syndromes, and limited physiological reserve may affect postoperative outcomes [20, 21]. CGA-based perioperative pathways can support optimization, prehabilitation, shared decision-making, and coordinated postoperative care [22, 23]; however, their implementation in oncological surgery remains uneven across institutions and countries, reflecting organizational barriers, workforce constraints, and resource limitations [24, 25].

To date, evidence on the development and implementation of geriatric (surgical) oncology across Europe remains scarce and fragmented [16, 26, 27]. This study, therefore, aims to provide a thorough overview of geriatricians’ involvement in the oncological and onco-surgical care of older patients across Europe, and to assess how far each country has progressed in implementing this type of care model. It also aims to identify educational needs and limitations, and to suggest solutions to improve geriatric oncology and geriatric surgical oncology in Europe.

## Methods

### Study design

In this descriptive, observational study, an anonymous online survey was distributed among geriatricians across Europe. The survey was designed in *Castor*, an electronic data capture platform, and consisted of multiple-choice and open-ended questions relating to the structure, members, and organizational practices of geriatric oncology units. No personal data were collected other than the participants’ profession, years of professional experience, and country of work.

### Survey development and setting

An English version of the survey was drafted and distributed to all principal investigators in the following European countries: the Netherlands, Spain, the United Kingdom, Norway, Denmark, Germany, France, Italy, Belgium, and Turkey. A panel of 15 experts in geriatric oncology assessed the face and content validity of the questionnaire. In addition, three of the principal investigators (PSM, IM, and HAPB) conducted a pilot test to evaluate clarity, comprehension, and completion time. During the pilot phase, each tester completed the full questionnaire and provided feedback on the wording of items, response options, the flow of the survey, its comprehensibility, and the estimated completion time. The coordinating team discussed the feedback and made minor changes. The final survey included 35 questions, structured into 3 sections: work and country details, current medical and surgical geriatric oncology practices within the respondent’s institution, and barriers for implementation and potential areas for improvement.

The original survey was written in English and translated into French and Turkish by native-speaking collaborators, at the request of national representatives (the different versions are provided as supplementary files 2–4).

### Dissemination strategy

The target population consisted of geriatricians and internal medicine specialists with specific geriatric training residing in European nations whose national geriatric medicine societies are members of the European Geriatrics Medicine Society (EuGMS). In addition to countries within strict geographic Europe, a few others were included due to their formal participation in EuGMS activities. Specifically, Israel was included as its national geriatric society is a EuGMS member, while Armenia, Belarus, Russia, and Northern Macedonia were also considered because their national societies participate in or observe EuGMS initiatives. Following expert validation, the final version of the survey was distributed via email through the EuGMS, the EuGMS Gero-Oncology and Perioperative Geriatric Medicine Special Interest Groups (SIGs), European national geriatric societies and the International Society of Geriatric Oncology (SIOG). Participating investigators also distributed the survey directly within their national networks. To maximize geographical spread and reduce center-specific recruitment, the survey was disseminated through broad national and international geriatric networks rather than selected centers. Because dissemination occurred through several overlapping networks, the exact number of eligible geriatricians reached in each country and the proportion of each national geriatric workforce represented by the respondents could not be determined. The survey was available from April 10 to May 23, 2025, with two reminder emails sent on April 22 and May 6, 2025.

### Data management

Data were collected and stored within the *Castor* platform. For confidentiality reasons, access to the raw data was restricted to two members of the research team (IM and HAPB). The survey was designed to be fully anonymous, and the responses were exported to an anonymized Excel spreadsheet. Because the survey was anonymous, it was not possible to identify individual co-author participation; therefore, any co-author who completed the survey and met the inclusion criteria would have been included in the respondent-level analysis. Similarly, because center-level identifiers were not collected, we could not exclude the possibility that multiple respondents came from the same institution. Each response was given a unique code before analysis to ensure confidentiality and facilitate data handling.

### Statistical analysis

A descriptive analysis was conducted for all survey responses. Categorical variables were presented as absolute frequencies (*n*) and percentages (%) where appropriate. Cross-tables were used to explore possible associations, including the Chi-squared test or Fisher’s exact test as appropriate, to test for significant associations. Subsequently, exploratory multivariable binary logistic regression analyses were performed to assess whether routine MDT participation remained associated with active involvement in treatment decision-making, provision of CGA-based recommendations addressing both CGA domains and oncological treatment, and positive feedback from oncology/surgery teams after adjustment for country group, institution type, and years of professional experience. Institution type was recoded as academic/university-affiliated versus non-academic institution, and years of professional experience were included as an ordinal variable. Country-specific coefficients were not interpreted, as the country group was included for adjustment only, and some country strata contained small numbers of respondents. Results are reported as adjusted odds ratios (aORs) with 95% confidence intervals.

For items that allow multiple responses, each response option was analyzed as a separate, non-mutually exclusive binary variable. Therefore, the percentages for these items may exceed 100%, and the findings should be interpreted as the reported presence of each category rather than as mutually exclusive responses.

All *p* values were two-sided, and a *p* value of less than 0.05 was considered statistically significant. An external statistician provided the initial statistical advice. PSM and HAPB performed the final data cleaning, statistical analyses, and interpretation using SPSS software, version 25.0 for Windows (IBM Corp., Armonk, NY, USA).

### Ethical considerations

As the study was descriptive in nature, the survey was anonymous, and no personally identifiable or health-related data was collected. Formal approval by an ethics committee was waived by the Medical Ethical Committee of Erasmus MC University Medical Center, Rotterdam, the Netherlands. Completing the survey implied informed consent.

## Results

### Study population

A total of 11,189 emails were distributed by the EuGMS to individual professionals, aiming to reach geriatricians in 41 countries. Of these, 2160 responses (19%) were received from 37 countries (90%). No responses were obtained from the Czech Republic, Latvia, Russia, or San Marino. Responses without information on the respondent’s country were excluded, yielding 1670 valid responses.

The average completion rate of the 2160 responses was 18%. We decided to include responses with < 100% completion of the survey, since participants may have stopped filling out the survey at a certain point due to a lack of structural geriatric oncology care, time constraints, or detailed information. Further evaluation of the responses revealed that those with ≤ 25% completion were almost exclusively from participants with other professional backgrounds, such as physiotherapists or neurologists. Furthermore, this group included three respondents who were residents in geriatric medicine, who were excluded since the survey focused on consultants. Therefore, we decided to include responses with a completion rate of at least 26% from geriatricians and/or specialists in internal medicine. Accordingly, responses from Armenia (one geriatrician with 23% survey progression), Cyprus (one physiotherapist with 19% progression), and North Macedonia (one neurologist) were excluded.

A total of 395 respondents with a completion rate of at least 26% from 34 countries were included in the final analysis. To simplify the presentation of the results in the tables, we analyzed all the respondents together (“all countries”). Furthermore, in-depth analyses of countries were performed for countries with ≥ 10 respondents to provide further insight. These countries included Italy, France, Spain, the Netherlands, Denmark, the United Kingdom, Turkey, Ireland, Portugal, and Belgium (see Fig. S1). The cut-off point of ≥ 10 responses per country was chosen to ensure adequate representation while maintaining clarity in the tables and figures. Supplementary File 1 provides a summary of the overall cross-country analysis of the 24 countries that were not included in the in-depth analysis.

### Survey respondents

Of the 395 respondents included, the majority of respondents were geriatricians (87%), 10% were internal medicine specialists focused on geriatrics, and 3% were both. In terms of their working experience in geriatrics, 22% of participants had worked as geriatrician for less than 5 years, 29% between 5 and 10 years, 29% between 11 and 20 years, and 20% more than 20 years.

Most respondents (88%) worked in general hospitals, whereas a minority of the respondents worked in geriatric hospitals (7%) and cancer centers (4%).

Regarding institution type, 57% were employed in university-affiliated or academic medical centers, 37% in national health service/public hospitals, and 5% in private or non-teaching hospitals.

Considering the structure of geriatric medicine services, 76% of respondents reported working within a dedicated geriatric medicine department. Meanwhile, 9% indicated the presence of geriatric beds without a full department, and 15% stated that their institutions had no dedicated geriatric unit.

### Geriatric oncology model screening and referral criteria for CGA

**Fig. 1:**
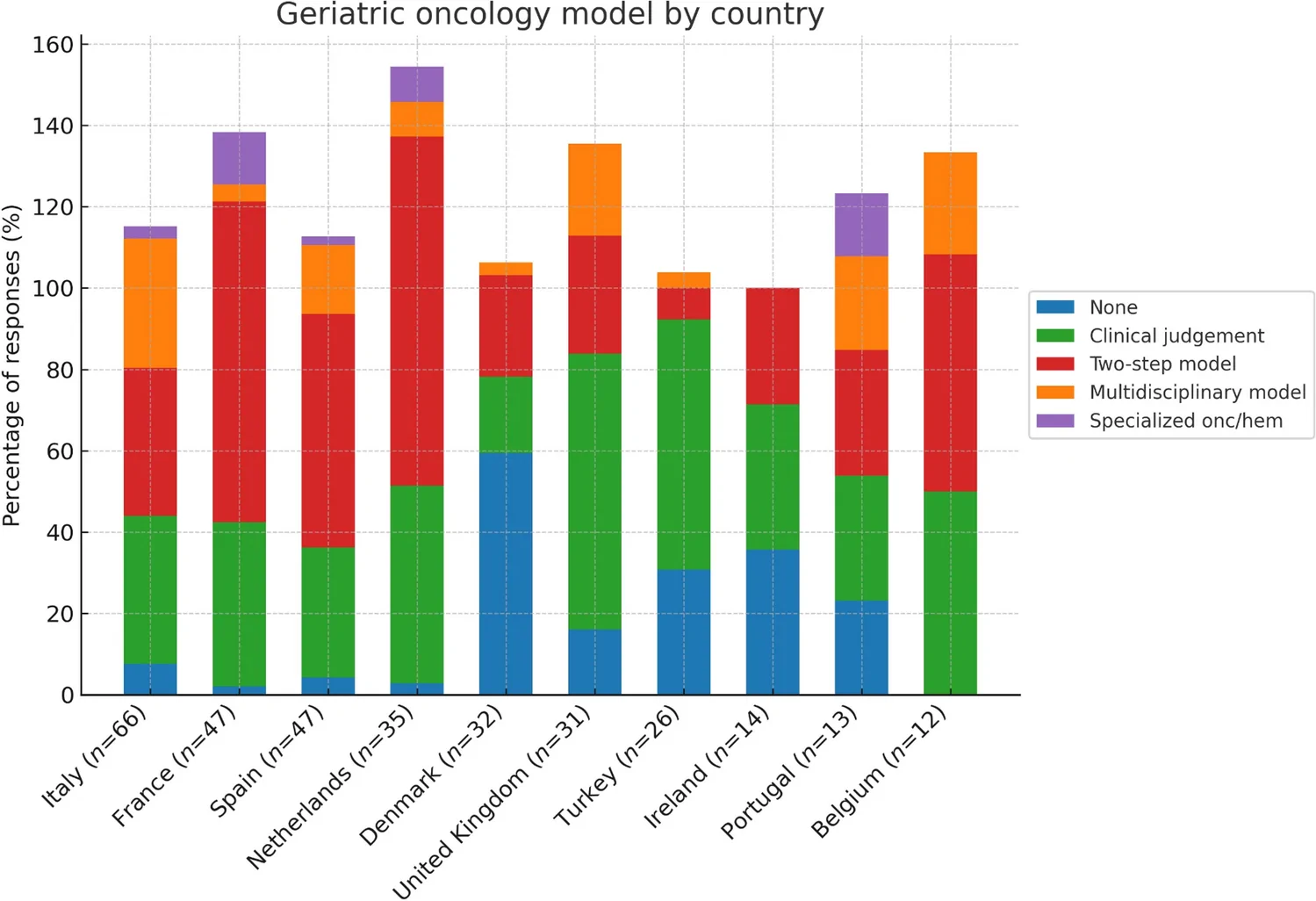
What geriatric oncology model exists in your country? Geriatric oncology model by country. Stacked bars show the percentage of respondents in each country reporting the organizational model(s) used for geriatric oncology care: none (patients are not referred), clinical judgment (patients are referred based upon clinical judgment of the referring physician), two-step model (referral to geriatrics in case of positive screening for frailty or an abnormal result according to an abbreviated GA), multidisciplinary model (a multidisciplinary consultative model in which a multidisciplinary team assesses all patients), and specialized onc/hem (there are oncologists and/or hematologists specialized in geriatrics). Sample sizes are indicated for each country on the *x*-axis. More than one answer option could be selected, so the summed percentages within a country may exceed 100%

As shown in Table 1 and Fig. 1, there was substantial heterogeneity in geriatric oncology practices across Europe. Of the 395 respondents, 19% reported no formal geriatric oncology model. The most common approaches were referral based on clinical judgment (43%) and a two-step model involving frailty screening followed by a CGA (42%). A smaller proportion (13%) reported using a multidisciplinary consultative model, while only 4% had oncologists or hematologists who specialized in geriatrics.

**Table 1:** Geriatric oncology models and practices for all countries, and by country (for 10 countries with the most respondents)

	All countries^1^ n = 395 (%)	Italy *n* = 66 (%)	France *n* = 47 (%)	Spain *n* = 47 (%)	The NL *n* = 35 (%)	Denmark *n* = 32 (%)	UK *n* = 31 (%)	Turkey* n* = 26 (%)	Ireland* n* = 14 (%)	Portugal *n* = 13 (%)	Belgium *n* = 12 (%)
What geriatric oncology model exists in your institution?*											
None. Patients are not referred	75 (19)	5 (8)	1 (2)	2 (4)	1 (3)	19 (55)	5 (16)	8 (31)	5 (36)	3 (23)	0
Patients are referred based upon clinical judgment of the referring physician	169 (43)	24 (36)	19 (40)	15 (32)	17 (49)	6 (19)	21 (68)	16 (62)	5 (36)	4 (31)	6 (50)
Two-step model: referral to geriatrics in case of positive screening for frailty or an abnormal result according to an abbreviated GA	166 (42)	24 (36)	37 (79)	27 (57)	30 (86)	8 (25)	9 (29)	2 (8)	4 (29)	4 (31)	7 (58)
A multidisciplinary consultative model: in which a multidisciplinary team assesses all patients	52 (13)	21 (32)	2 (4)	8 (17)	3 (9)	1 (3)	7 (23)	1 (4)	0	3 (23)	3 (25)
There are oncologists and/or hematologists specialized in geriatrics	14 (4)	2 (3)	6 (13)	1 (2)	3 (9)	0	0	0	0	2 (15)	0
What are the referral criteria to the geriatrician?*											
Age	154 (39)	28 (42)	28 (60)	16 (34)	4 (11)	4 (13)	8 (26)	14 (54)	5 (36)	6 (46)	7 (58)
Positive screening for frailty screening test or other GA screening test	250 (63)	39 (59)	43 (92)	36 (77)	32 (91)	14 (44)	22 (71)	10 (39)	7 (50)	5 (39)	8 (67)
Other^2^	70 (18)	6 (9)	6 (13)	8 (17)	10 (29)	4 (13)	12 (39)	5 (19)	5 (36)	3 (23)	0
Which screening scales are used to decide whether or not to refer a patient?*											
Clinical Frailty Scale (CFS)	125 (32)	23 (35)	3 (6)	10 (21)	5 (14)	13 (41)	22 (71)	12 (46)	6 (43)	5 (39)	0
Eastern Cooperative Oncology Group (ECOG) score	40 (10)	11 (17)	1 (2)	8 (17)	0	0	2 (7)	9 (35)	3 (21)	0	1 (8)
Geriatric 8 (G8)	197 (50)	34 (52)	44 (94)	38 (81)	29 (83)	5 (16)	3 (10)	5 (19)	3 (21)	8 (62)	10 (83)
Vulnerable Elders Survey (VES-13)	13 (3)	3 (5)	0	4 (9)	0	0	0	0	0	0	0
Geriatric Assessment in Hematology (GAH)	5 (1)	0	0	3 (6)	1 (3)	0	0	1 (4)	0	0	0
The Identification of Seniors At Risk-Hospitalized Patients (ISAR-HP)	7 (2)	0	0	1 (2)	0	0	0	1 (4)	0	0	2 (17)
Edmonton Frail Scale (EFS)	11 (3)	2 (3)	0	0	1 (3)	0	2 (7)	1 (4)	0	0	1 (8)
Groningen Frailty Indicator (GFI)	8 (2)	0	0	0	7 (20)	0	0	0	0	0	0
Triage Risk Screening Tool (TRST)	6 (2)	3 (5)	0	2 (4)	0	0	0	0	0	0	0
The Fried frailty phenotype (FP)	13 (3)	5 (8)	2 (4)	3 (6)	0	0	1 (3)	2 (8)	0	0	0
The Barber test (BT)	1 (< 1)	0	0	0	0	0	0	0	0	0	0
Cognitive screening 6CIT	14 (4)	0	0	1 (2)	7 (20)	0	2 (7)	1 (4)	1 (7)	1 (8)	0
Cognitive screening Mini-COG	32 (8)	10 (15)	0	6 (13)	3 (9)	0	1 (3)	2 (8)	0	2 (15)	0
Other^3^	62 (16)	7 (11)	7 (15)	3 (6)	6 (17)	7 (22)	9 (29)	3 (12)	3 (21)	1 (8)	0
At what stage are patients referred to geriatricians?*											
At diagnosis	180 (46)	27 (41)	41 (87)	24 (51)	27 (77)	4 (13)	16 (52)	4 (15)	2 (14)	6 (46)	9 (75)
At start of oncological treatment	163 (41)	31 (47)	27 (57)	25 (53)	15 (43)	4 (13)	13 (42)	5 (19)	3 (21)	4 (31)	7 (58)
After the start of oncological treatment	72 (18)	11 (17)	15 (32)	11 (23)	2 (6)	2 (6)	9 (29)	5 (19)	3 (21)	2 (15)	1 (8)
Refractory or recurrent disease	76 (19)	14 (21)	17 (36)	12 (26)	1 (3)	1 (3)	10 (32)	2 (8)	1 (7)	1 (8)	1 (8)
Once decision made for palliative treatment o	91 (23)	22 (33)	7 (15)	8 (17)	1 (3)	5 (17)	12 (39)	15 (58)	1 (7)	1 (8)	2 (17)
When complications/toxicity of treatment occurs	95 (24)	18 (27)	14 (30)	10 (21)	4 (11)	5 (16)	13 (42)	9 (35)	1 (7)	3 (23)	2 (17)
Other^4^	29 (7)	6 (9)	0	1 (2)	1 (3)	7 (22)	10 (32)	1 (4)	4 (29)	4 (31)	0
*Based on CGA, are recommendations made?*^5^											
Only conclusion	42 (13)	10 (18)	1 (2)	2 (5)	0	4 (25)	4 (15)	4 (16)	1 (14)	1 (8)	1 (8)
Conclusions and treatment recommendations only for the oncological treatment	14 (4)	2 (4)	0	1 (2)	0	2 (13)	1 (4)	3 (12)	0	1 (8)	1 (8)
Conclusions and treatment recommendations only for optimization of CGA domains	123 (37)	19 (33)	10 (23)	10 (24)	2 (6)	8 (50)	14 (52)	15 (60)	4 (57)	6 (50)	4 (33)
Conclusions and treatment recommendations for optimization of CGA domains and for oncological treatment	158 (47)	26 (47)	33 (75)	29 (69)	30 (94)	2 (13)	8 (30)	3 (12)	2 (29)	4 (33)	6 (50)
How are CGA conclusions and recommendations shared?*											
Reported in a multidisciplinary team meeting	105 (27)	26 (39)	9 (19)	17 (36)	24 (67)	1 (3)	5 (16)	2 (8)	0	6 (46)	3 (25)
By phone	58 (15)	4 (6)	14 (30)	6 (13)	15 (43)	2 (6)	3 (10)	3 (11)	0	2 (15)	3 (25)
A written summary in the patient file	268 (69)	38 (58)	41 (87)	34 (72)	28 (80)	13 (41)	22 (71)	20 (77)	6 (43)	7 (54)	12 (100)
Other^6^	33 (8)	1 (2)	7 (15)	2 (4)	5 (14)	2 (6)	7 (23)	2 (8)	2 (14)	1 (8)	0

Percentages are rounded to whole numbers; therefore, totals may not sum exactly to 100% and values < 1% are shown as < 1%

*CGA* comprehensive geriatric assessment, *NL* Netherlands, *UK* United Kingdom

*Respondents could select one or more options

^1^All countries included all respondents, geriatricians, and/or internists with a focus on geriatrics, with ≥ 26% progression

^2^Among the options provided, the most frequent is clinical judgment. Some also combine age with frailty or complexity, while others add the presence of geriatric syndromes such as polypharmacy, malnutrition, or cognitive impairment. The need for palliative care is also mentioned twice. Many respondents state that there are no referral criteria

^3^Many respondents state that they do not use any scale or rely on clinical judgment. Others mention the use of: VIG-Frail, Rockwood CFS, FiCGA, Tinetti, 4AT, clock drawing test, ADL, IADL, MMSE, MNA, Timed Up and Go, EORTC, and FOG (Filtre Onco-Gériatrique)

^4^Many respondents write that patients can be referred at any of these points; others mention at MDT, before surgery, or when palliative care is needed. Several describe that patients are not referred, or that it does not apply

^5^Percentages exclude missing values. Missing by country (not included in %): All *n* = 58, Italy *n* = 9, France *n* = 3, Spain *n* = 5, The Netherlands *n* = 3, Denmark *n* = 16, United Kingdom *n* = 4, Turkey *n* = 1, Ireland *n* = 7, Portugal *n* = 1, Belgium *n* = 0

^6^Many state that it is done by email; one mentions that in a project setting, the conclusion is written in a medical journal. Many others report that it does not apply

Figure 1 illustrates the marked between-country variation in reported models. In this figure, models could overlap within countries, because multiple responses were allowed. Structured two-step models were most frequently reported in several Western European countries. Referral criteria were primarily based on frailty screening (63%), though 39% relied on chronological age, particularly in Italy (42%) and France (60%). Out-of-depth analyses suggested that age-based referral was common in several countries with small numbers of respondents (≤ 3 per country), including Austria and Sweden, as well as some Eastern European countries, such as Romania and Poland. The G8 frailty screening tool was the most commonly used pre-CGA screening instrument to guide referral to geriatrics (50%), followed by the Clinical Frailty Scale (32%) and the Eastern Cooperative Oncology Group Performance Status Scale (ECOG-PS) (10%). The use of the G8 was almost universal in France (94%) and predominant in the Netherlands (83%) and Spain (81%). In contrast, the Clinical Frailty Scale was predominantly used in the UK (71%) (see Table 1).

**Fig. 2:**
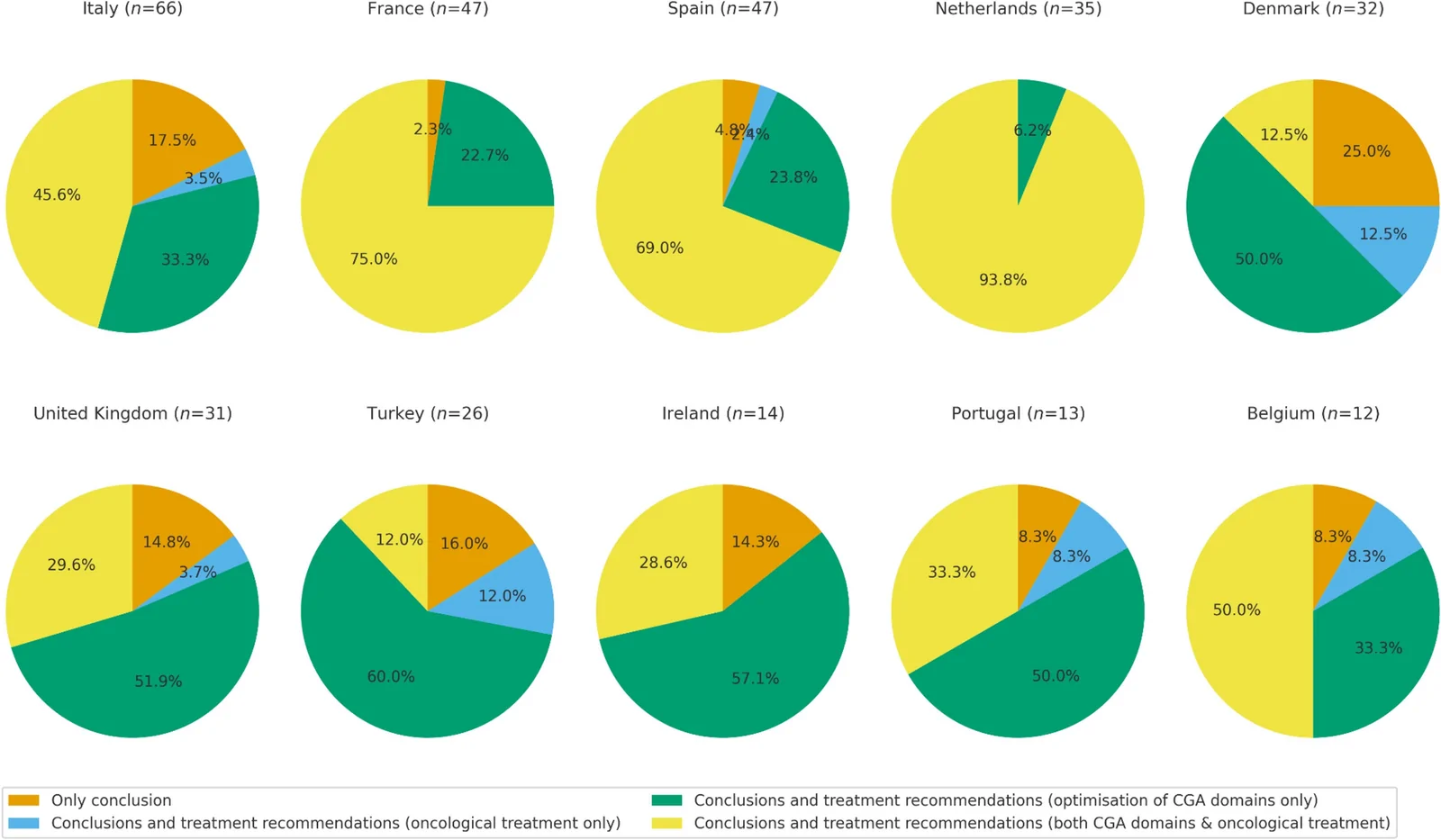
CGA conclusions and recommendations per country. CGA conclusions and recommendations per country. Pie charts show, for each country, the proportion of respondents describing how comprehensive geriatric assessment (CGA) findings are translated into clinical output. Four response categories are displayed: only conclusion, conclusions and treatment recommendations only for the oncological treatment, conclusions and treatment recommendations only for optimization of CGA domains, and conclusions and treatment recommendations for both CGA domains and oncological treatment. Sample size for each country is given in the panel title. More than one answer option could be selected, so percentages within a country may exceed 100%. Percentages are presented with one decimal place

Patients were mostly referred at the time of diagnosis (46%) or at the start of oncological treatment (41%). Referral at diagnosis was especially frequent in France (87%) and the Netherlands (77%), whereas referral at the start of treatment was also common in Spain (53%) and Italy (47%). CGA was primarily conducted by geriatricians (70%), and nurse specialists were also involved in 22% of respondents. Figure 2 shows marked country-level variation in the scope of CGA outputs, with combined recommendations addressing both CGA-domain optimization and oncological treatment reported most frequently in some countries, particularly the Netherlands, France, and Spain. CGA findings were most commonly shared through written summaries in the patient record, while discussion in MDT meetings was less frequent.

Routine follow-up was uncommon (17%), though 47% of respondents provided follow-up if requested. Regular follow-up was most prevalent in Spain (43%) and Italy (29%). When considering all countries, routine follow-up was reported in Belarus (100%, 1 respondent), followed by Israel (67%, 2 of 3 respondents) and Sweden (50%, 1 of 2 respondents).

### CGA output and multidisciplinary team participation

As shown in Table 2, many respondents reported participating in multidisciplinary team (MDT) meetings, most often oncogeriatric meetings (30%), followed by occasional (18%) and pre-surgery (5%) meetings. Routine participation was highest in the Netherlands (54%) and Spain (53%). Attendance of specific oncological MDT meetings was highest for colorectal (23%), breast (15%), upper gastrointestinal (13%), and prostate and lung cancer (both 12%), particularly in Southern Europe. Further analysis of countries with < 10 respondents showed that respondents from Belarus (100%, 1 respondent), Hungary (100%, 1 respondent) and Finland (57%, 4 respondents) also routinely participated in MDT meetings.

**Table 2:** Multidisciplinary geriatric oncology practices for all respondents, and by country

	All countries^1^ *n* = 395 (%)	Italy *n* = 66 (%)	France *n* = 47 (%)	Spain *n* = 47 (%)	The NL *n* = 35 (%)	Denmark *n* = 32 (%)	UK *n* = 31 (%)	Turkey* n* = 26 (%)	Ireland* n* = 14 (%)	Portugal *n* = 13 (%)	Belgium *n* = 12 (%)
Do you take part in multidisciplinary oncological meetings?*											
Yes, in casual (incidental) meetings	69 (18)	14 (21)	14 (30)	8 (17)	8 (23)	0	4 (13)	3 (12)	1 (7)	2 (15)	5 (42)
Yes, routinely in oncogeriatric meetings	120 (30)	25 (38)	14 (30)	25 (53)	19 (54)	0	6 (19)	2 (8)	0	5 (39)	3 (25)
Yes, routinely in pre-surgery meetings	21 (5)	1 (2)	1 (2)	5 (11)	9 (26)	0	3 (10)	0	0	0	0
No, I do not take part in multidisciplinary oncological meetings	159 (40)	14 (21)	19 (40)	12 (26)	4 (11)	15 (47)	18 (58)	20 (77)	5 (36)	5 (39)	4 (33)
Which tumor-specific meetings do you attend?*											
Bladder cancer	37 (9)	7 (11)	9 (19)	8 (17)	4 (11)	0	1 (3)	1 (4)	0	0	3 (25)
Breast cancer	59 (15)	24 (36)	11 (23)	9 (19)	5 (14)	0	2 (7)	0	0	4 (31)	1 (8)
Colorectal cancer	91 (23)	14 (21)	17 (36)	24 (51)	18 (51)	1 (3)	2 (7)	0	0	1 (8)	4 (33)
Head and neck cancer	23 (6)	5 (8)	3 (6)	3 (6)	8 (23)	0	1 (3)	1 (4)	0	0	0
Hematological cancer	35 (9)	11 (17)	8 (17)	9 (19)	2 (6)	0	1 (3)	0	0	0	1 (8)
Hepato-biliary cancer	36 (9)	7 (11)	9 (19)	6 (13)	5 (14)	0	0	0	0	0	3 (25)
Kidney (renal cell) cancer	24 (6)	5 (8)	7 (15)	4 (9)	2 (6)	0	0	0	0	0	2 (17)
Lung cancer	48 (12)	13 (20)	11 (23)	10 (21)	4 (11)	0	3 (10)	0	0	1 (8)	1 (8)
Neuro-oncology	11 (3)	3 (5)	2 (4)	3 (6)	0	0	0	0	0	0	0
Orthopedic oncology	8 (2)	2 (3)	1 (2)	1 (2)	1 (3)	0	1 (3)	0	0	0	0
Pancreatic cancer	40 (10)	6 (9)	11 (23)	8 (17)	5 (14)	0	0	0	0	0	2 (17)
Prostate cancer	49 (12)	14 (21)	11 (23)	13 (28)	3 (9)	0	1 (3)	1 (4)	0	0	3 (25)
Skin cancer	12 (3)	3 (5)	3 (6)	1 (2)	4 (11)	0	0	0	0	0	0
Upper GI-tract cancer	50 (13)	9 (14)	11 (23)	8 (17)	10 (29)	0	3 (10)	0	0	1 (8)	2 (17)
Gynecological cancer	37 (9)	9 (14)	11 (23)	10 (21)	3 (9)	0	1 (3)	0	0	0	0
Other^2^	5 (1)	1 (2)	2 (4.3)	0	4 (11)	0	1 (3)	0	0	0	0
Not applicable	185 (47)	15 (23)	26 (55)	12 (26)	8 (23)	14 (44)	20 (65)	23 (89)	6 (43)	7 (54)	6 (50)
*Do you participate in treatment decision-making?*^3^											
Yes	66 (20)	7 (13)	13 (30)	13 (31)	14 (45)	1 (7)	5 (19)	1 (4)	0	2 (17)	2 (18)
In some cases	159 (49)	36 (69)	26 (59)	19 (45)	16 (52)	4 (27)	10 (37)	7 (28)	2 (33)	7 (58)	6 (55)
No	101 (31)	9 (17)	5 (11)	10 (24)	1 (3)	10 (67)	12 (44)	17 (68)	4 (67)	3 (25)	3 (27)
*Do you get positive feedback from oncology/surgery teams?*^4^											
Yes	137 (42)	24 (46)	23 (54)	23 (56)	17 (55)	7 (47)	9 (33)	6 (24)	2 (33)	5 (42)	6 (55)
Sometimes	119 (37)	21 (40)	17 (40)	17 (42)	14 (45)	3 (20)	8 (30)	8 (32)	2 (33)	4 (33)	4 (36)
No	68 (21)	7 (14)	3 (7)	1 (2)	0	5 (33)	10 (37)	11 (44)	2 (33)	3 (25)	1 (9)

Percentages are rounded to whole numbers; therefore, totals may not sum exactly to 100% and values < 1% are shown as < 1%

*CGA* comprehensive geriatric assessment, *GI-tract* gastrointestinal tract, *NL* Netherlands, *UK* United Kingdom

*Respondents could select one or more options

^1^All countries included all respondents, geriatricians and/or internists with a focus on geriatrics, with ≥ 26% progression

^2^They add sarcomas and neuroendocrine tumors

^3^Percentages exclude missing values. Missing by country (not included in %): All *n* = 69, Italy *n* = 14, France *n* = 3, Spain *n* = 5, The Netherlands *n* = 4, Denmark *n* = 17, United Kingdom *n* = 4, Turkey *n* = 1, Ireland *n* = 8, Portugal *n* = 1, Belgium *n* = 1

^4^Percentages exclude missing values. Missing by country (not included in %): All *n* = 71, Italy *n* = 14, France *n* = 4, Spain *n* = 6, The Netherlands *n* = 4, Denmark *n* = 17, United Kingdom *n* = 4, Turkey *n* = 1, Ireland *n* = 8, Portugal *n* = 1, Belgium *n* = 1

**Fig. 3:**
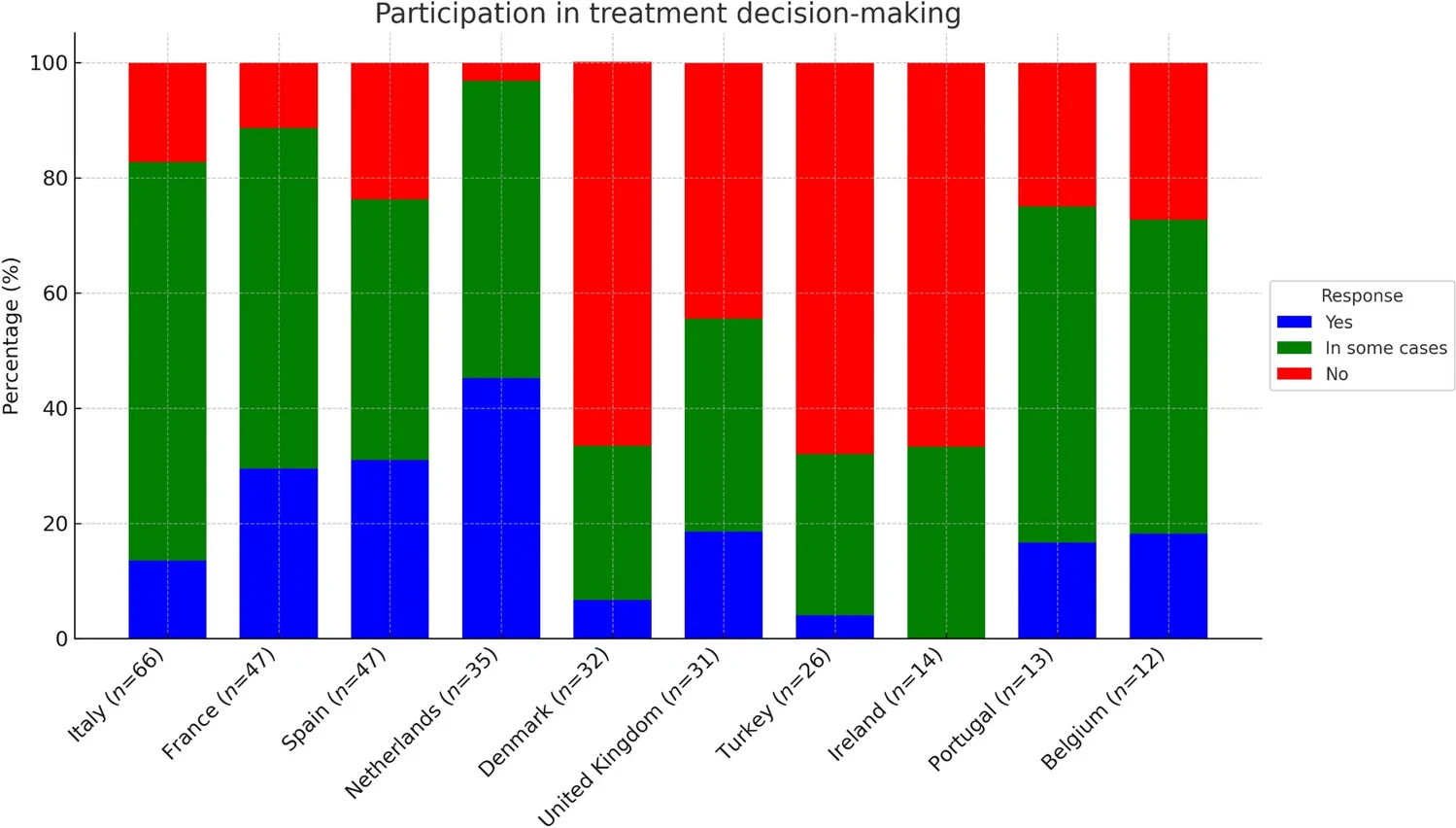
Participation in decision-making per country. Participation in treatment decision-making per country. Stacked bars show, for each country, the percentage of respondents indicating whether geriatric assessment/CGA findings are incorporated into cancer treatment decision-making: yes (systematically used for decisions), in some cases (used selectively depending on the patient or setting), or no (not used for decision-making). Sample size for each country is indicated on the *x*-axis. Only one answer option could be selected, therefore percentages within each country sum to 100%

In terms of treatment decision-making, geriatricians were involved either routinely (20%) or on a case-by-case basis (49%). The remaining 31% were not involved in oncological or surgical treatment decisions. All respondents from Belarus (*n* = 1), Croatia (*n* = 1), Hungary (*n* = 1), Slovakia (*n* = 1), and Sweden (*n* = 2) participated in treatment decision-making. The in-depth analysis of the countries with ≥ 10 respondents revealed that the highest levels of overall involvement (including “yes” and “in some cases”) were observed in the Netherlands (97%), France (89%), and Italy (83%) (see Table 2). Figure 3 further illustrates the between-country variation in treatment decision-making, with higher involvement reported in the Netherlands, France, and Italy, and lower involvement in Denmark, Turkey, and Ireland.

### Associations between MDT participation, CGA output, and participation in decision-making, and receiving positive reward from oncologists and surgeons

Routine participation in MDT meetings was strongly associated with both involvement in oncological treatment decision-making and the CGA-based recommendations (*p* < 0.001). Among respondents who routinely attended MDT meetings (*n* = 85), 47% reported actively participating in treatment decisions, compared to just 11% of those who did not attend. Similarly, 77% of MDT participants provided recommendations addressing both CGA-domain optimization and oncological treatment, compared to 37% of non-MDT participants.

In exploratory multivariable logistic regression adjusted for country, institution type, and years of professional experience, routine MDT participation remained associated with active involvement in treatment decision-making (aOR 6.01, 95% CI 3.08–11.7, *p* < 0.001) and with provision of CGA-based recommendations addressing both CGA domains and oncological treatment (aOR 5.81, 95% CI 3.16–10.7, *p* < 0.001).

Furthermore, positive feedback from oncologists and surgeons regarding geriatric input was consistently associated with MDT attendance and the incorporation of CGA-driven treatment recommendations (*p* < 0.001). Among respondents who routinely attended MDT meetings (*n* = 84), the majority (74%) reported receiving positive feedback from oncology or surgery colleagues. By contrast, among those who did not routinely attend MDT meetings (*n* = 240), positive feedback was reported by 31% of respondents. Of these, 41% said it was only sometimes provided and 28% said they received no positive feedback. In the adjusted logistic regression model, routine MDT participation remained associated with positive feedback from oncology/surgery teams (aOR 3.75, 95% CI 1.96–7.14, *p* < 0.001).

### Geriatric surgical oncology models

As shown in Table 3, 38.5% of all respondents reported being involved in co-managing hospitalized surgical oncology patients, while 61.5% were not. Regarding collaboration with surgical services, more than half indicated that there was no structural collaboration. The most common structure was a liaison model, where geriatricians were consulted on specific cases, such as delirium or previous pre-surgery CGA. A smaller proportion (9%) described a co-management model in which geriatricians are structurally present on the surgical ward.

**Table 3:** Geriatric surgery models: co-management for hospitalized surgical patients for all respondents, and by country

	All countries^1^ n = 395 (%)	Italy *n* = 66(%)	France *n* = 47 (%)	Spain *n* = 47 (%)	The NL *n* = 35 (%)	Denmark *n* = 32 (%)	UK *n* = 31 (%)	Turkey* n* = 26 (%)	Ireland* n* = 14 (%)	Portugal *n* = 13 (%)	Belgium *n* = 12 (%)
*Are you involved in the management of patients during hospitalization for oncological surgery?*^2^											
Yes	122 (39)	15 (30)	20 (47)	17 (42)	19 (63)	8 (57)	11 (41)	4 (17)	2 (33)	7 (58)	6 (55)
No	195 (62)	35 (70)	23 (54)	24 (59)	11 (37)	6 (43)	16 (59)	20 (83)	4 (67)	5 (42)	5 (46)
*Do you have a structural form of collaboration with oncological surgery?*^3^											
Yes, a liaison model (the geriatrician is asked for advice and/or co-management for specific patients with, e.g., a delirium, or for patients who received a pre-surgery CGA)	116 (37)	18 (36)	21 (49)	17 (42)	19 (63)	6 (43)	12 (44)	3 (13)	0	2 (17)	8 (73)
Yes, a co-management model (the geriatrician is structurally present at the surgical ward for advice and/or co-management)	28 (9)	3 (6)	0	7 (17)	1 (3)	2 (14)	4 (1)	1 (4.2)	0	2 (17)	0
Yes, other^4^	1 (< 1)	0	1 (2)	0	0	0	0	0	0	0	0
No	172 (54)	29 (58)	21 (49)	17 (42)	10 (33)	6 (43)	11 (41)	20 (83)	6 (100)	8 (67)	3 (27)
For which tumor types are you involved during hospitalization?*											
Bladder cancer	58 (1%)	9 (14)	8 (17)	9 (19)	7 (20)	5 (16)	7 (23)	1 (4)	1 (7)	1 (8)	0
Breast cancer	36 (9)	7 (11)	5 (11)	7 (15)	2 (6)	1 (3)	7 (23)	1 (4)	1 (7)	2 (15)	0
Colorectal cancer	105 (27)	11 (17)	16 (34)	19 (40)	16 (46)	6 (19)	11 (36)	2 (8)	1 (7)	3 (23)	0
Head and neck cancer	29 (7)	2 (3)	5 (11)	2 (4)	7 (20)	0	3 (10)	1 (4)	1 (7)	1 (8)	0
Hematological cancer	25 (6)	5 (8)	4 (9)	7 (15)	4 (11)	1 (3)	1 (3)	0	1 (7)	0	0
Hepato-biliary cancer	43 (11)	6 (9)	7 (15)	8 (17)	4 (11)	2 (6)	3 (10)	2 (8)	1 (7)	1 (8)	0
Kidney (renal cell) cancer	40 (10)	4 (6)	7 (15)	4 (9)	7 (20)	2 (6)	6 (19)	0	1 (7)	0	0
Lung cancer	35 (9)	5 (8)	7 (15)	7 (15)	3 (9)	0	5 (16)	3 (12)	1 (7)	0	0
Neuro-oncology	14 (4)	2 (3)	2 (4)	2 (4)	1 (3)	0	2 (7)	1 (4)	1 (7)	0	0
Orthopedic oncology	19 (5)	1 (2)	1 (2)	3 (6)	3 (9)	0	4 (13)	2 (8)	1 (7)	0	0
Pancreatic cancer	51 (13)	4 (6)	13 (28)	9 (19)	6 (17)	5 (16)	2 (7)	2 (8)	1 (7)	1 (8)	0
Prostate cancer	50 (13)	8 (12)	7 (15)	9 (19)	2 (6)	4 (13)	5 (16)	2 (8)	1 (7)	1 (8)	0
Skin cancer	16 (4)	0	6 (13)	0	3 (9)	0	3 (10)	0	1 (7)	0	0
Upper GI-tract cancer	63 (16)	9 (14)	11 (23)	8 (17)	9 (26)	4 (13)	9 (29)	2 (8)	1 (7)	3 (23)	0
Gynecological cancer	43 (11)	5 (8)	9 (19)	10 (21)	5 (14)	1 (3)	3 (10)	1 (4)	1 (7)	1 (8)	0
Other^5^	3 (< 1)	0	0	1 (2)	3 (9)	1 (3)	1 (3)	0	1 (7)	1 (8)	0
Not applicable	184 (47)	32 (49)	25 (53)	19 (40)	10 (29)	6 (19)	14 (45)	21 (81)	5 (36)	7 (54)	3 (75)
*Do you offer a prehabilitation program?*^6^											
Yes	173 (53)	24 (45)	27 (63)	35 (85)	21 (68)	2 (13)	10 (37)	12 (48)	3 (50)	5 (39)	4 (36)
No	127 (39)	25 (47)	13 (30)	4 (10)	9 (29)	12 (80)	11 (41)	8 (32)	1 (17)	8 (62)	6 (55)
I do not know	26 (8)	4 (8)	3 (7)	2 (5)	1 (3)	1 (7)	6 (22)	5 (20)	2 (33)	0	1 (9)
What does the prehabilitation program consist of?*											
Exercise	163 (41)	21 (32)	25 (53)	35 (75)	20 (57)	2 (6)	10 (32)	10 (39)	3 (21)	5 (39)	4 (33)
Nutrition	161 (41)	23 (35)	26 (55)	35 (75)	20 (57)	2 (6)	8 (2)6	10 (39)	1 (7)	5 (39)	4 (33)
Psychological	87 (22)	14 (21)	17 (36)	16 (34)	8 (23)	0	4 (13)	9 (35)	0	2 (15)	2 (17)
Quit smoking/alcohol program	48 (12)	8 (12)	2 (4)	9 (19)	11 (31)	2 (6)	4 (13)	3 (12)	0	2 (15)	0
Other^7^	17 (4)	1 (2)	3 (6)	4 (9)	1 (3)	0	1 (3)	2 (8)	0	1 (8)	0

Percentages are rounded to whole numbers; therefore, totals may not sum exactly to 100% and values < 1% are shown as < 1%

*UK* United Kingdom

*Respondents could select one or more options

^1^All countries included all respondents, geriatricians and/or internists with a focus on geriatrics, with ≥ 26% progression

^2^Percentages exclude missing values. Missing by country (not included in %): All *n* = 78, Italy *n* = 16, France *n* = 4, Spain *n* = 6, The Netherlands *n* = 5, Denmark *n* = 18, United Kingdom *n* = 4, Turkey *n* = 2, Ireland *n* = 8, Portugal *n* = 1, Belgium *n* = 1

^3^Percentages exclude missing values. Missing by country (not included in %): All *n* = 78, Italy *n* = 16, France *n* = 4, Spain *n* = 6, The Netherlands *n* = 5, Denmark *n* = 18, United Kingdom *n* = 4, Turkey *n* = 2, Ireland *n* = 8, Portugal *n* = 1, Belgium *n* = 1

^4^Some mention the possibility of two coexisting models. In other cases, geriatricians are consulted if frailty, complications, or the need for palliative care

^5^Several clarify that all options are possible depending on the patient’s needs, the presence of geriatric syndromes, etc.

^6^Percentages exclude missing values. Missing by country (not included in %): All *n* = 69, Italy *n* = 13, France *n* = 4, Spain *n* = 6, The Netherlands *n* = 4, Denmark *n* = 17, United Kingdom *n* = 4, Turkey *n* = 1, Ireland *n* = 8, Portugal *n* = 1, Belgium *n* = 1

^7^They include the management of anemia, medication review with deprescription, sleep hygiene measures, home assessment for early discharge, as well as social and family support. One person emphasizes that this is part of the CGA

In terms of tumor types, involvement during hospitalization was most frequent for colorectal cancer (27%), followed by upper gastrointestinal (16%) and bladder (15%) cancers.

At the country level, the Netherlands (63%) and Denmark (57%) showed the highest overall involvement of geriatricians in surgical oncology, followed by Belgium (55%) and Portugal (58%). Liaison models were predominant in most countries, particularly in the Netherlands (63%), France (49%), and Spain (42%), whereas co-management models were less frequent but more established in Spain (17%) and Denmark (14%). Taking into account all countries, the respondents from Hungary (*n* = 1) and Slovakia (*n* = 1) confirmed their involvement in surgical oncology through a liaison model.

France, Spain, and the Netherlands reported broader engagement across multiple tumor sites, particularly colorectal, upper gastrointestinal, and pancreatic cancers. In contrast, Turkey, Ireland, Belgium, Portugal, and the UK participated only in a narrow range of tumor types, most often isolated cases of colorectal or breast cancer (see Table 3).

Just over half of the respondents (53%) reported offering a prehabilitation program for surgical oncology patients. The most common components among centers were exercise training (41%) and nutritional support (41%), followed by psychological interventions (22%).

### Education and national guidelines

Almost half of the respondents (48%) reported that they had no structured educational system in place. The first analysis accordingly revealed that Albania (*n* = 1), Bulgaria (*n* = 1), Iceland (*n* = 4), Malta (*n* = 3), and Slovakia (*n* = 1) reported a lack of an educational system in 100% of responses. Similarly, all respondents from these countries declared that they did not have specific treatment guidelines for older patients with cancer. Overall, 23% of respondents chose the same answer. Contradictory answers were seen for both themes for the rest of the participating countries. Notably, contrasting answers were given regarding the education system (none vs. any kind vs. I do not know) for Italy (50% vs. 45% vs. 4%) and the Netherlands (40% vs. 71% vs. 0%), as well as regarding treatment guidelines (none + clinical recommendations only vs. yes vs. I do not know) for Italy (38% vs 53% vs 9%) and Spain (51% vs 45% vs 4%).

### Limitations, solutions, and needs for further development of geriatric oncology and geriatric oncological surgery

Supplementary Table S1 details the reported limitations, proposed solutions, and priority needs for the further development of geriatric oncology and geriatric oncological surgery across Europe. Respondents identified several barriers to developing geriatric oncology models across Europe. The most frequently reported limitations were a lack of engagement from other medical specialists (47%), a shortage of geriatricians (44%), and an insufficient education in geriatric oncology (42%). Other barriers reported included limited time and staff resources, insufficient funding, poor communication between professionals, and a lack of awareness of policies.

Similar constraints were identified in surgical geriatric oncology, including a lack of collaboration with surgeons and oncologists (44%), a shortage of geriatricians (42%), and a lack of specific education (37%).

Proposed solutions primarily focused on education and training for geriatricians, as well as for oncological and surgical specialists. Around 45–50% of respondents supported the implementation of dual training or fellowship programs. Many also supported regulatory measures (42%) and advocacy toward policymakers (40%) to improve institutional and political support. Finally, over 80% of respondents indicated their willingness to attend a masterclass on perioperative geriatric medicine in case this would be established in future.

## Discussion

This European survey highlights significant differences in the organization and integration of geriatric oncology and geriatric oncological surgery throughout Europe. Rather than identifying a single dominant model, respondents reported a variety of referral methods based on clinical judgment, two-step screening/CGA pathways, and, in some settings, no formal geriatric oncology model. Integration within MDTs and perioperative oncological surgery pathways was also variable. Exploratory analyses showed that routine MDT participation was associated with active involvement in treatment decision-making, provision of broader CGA-based recommendations, and positive feedback from oncology/surgery teams. The surgical oncology findings were particularly notable, with limited structured collaboration and a predominance of liaison models over co-management models. Together, these findings suggest that the main challenge lies not only in determining whether geriatric assessments are available but also in ensuring their consistent integration within oncology and oncological surgery pathways.

This Europe-wide survey shows substantial heterogeneity observed in screening strategies, referral pathways, and CGA implementation across countries. Some health systems, notably France, have been developing structured geriatric oncology models for more than 2 decades, supported by sustained collaboration between oncologists and geriatricians [28]. The creation of the Pilot Oncogeriatric Coordination Units (UPCOGs) by the French National Cancer Institute (INCA) [29, 30] in 2005 represents one of the earliest national initiatives to formalize geriatric oncology pathways, expand professional training, and promote systematic multidisciplinary consultation. [29]. Evidence from other European countries has underscored similar challenges, particularly the limited uptake of CGA and the structural barriers hindering its routine implementation. Belgian and Dutch surveys have repeatedly demonstrated low rates of GA implementation by treating physicians and persistent barriers such as high workload, limited staffing, and insufficient time or financial resources [24, 31–33]. In Spain, a national questionnaire revealed that many older patients were referred primarily to palliative care services rather than geriatricians, and that guideline use or geriatric screening tools were uncommon despite clinicians recognizing the need for tailored, person-centered care [27]. International data from SIOG, the earliest dating back to 2007 [15] and the most recent global report [34], reinforce the fact that although geriatric oncology is recognized as an essential discipline, its clinical integration remains fragmented due to resource constraints, lack of training opportunities, and limited institutional support. An interview study exploring geriatric oncology practice in five European cancer centers published in 2024 reported similar findings, highlighting considerable variability in CGA components, resource allocation, multidisciplinary communication, and implementation models, despite unanimous agreement on the benefits of interdisciplinary collaboration [16].

The association observed between MDT participation, involvement in treatment decision-making, and the delivery of CGA-based oncological treatment recommendations suggests that formal geriatric input and geriatrician ‘membership’ of the MDT may be an important component of integrated multidisciplinary cancer care. These findings are consistent with existing literature demonstrating that MDTs facilitate more personalized and evidence-based treatment planning, particularly when the patient priorities, functional status, comorbidities, and social circumstances of older adults are considered, as evaluated through the CGA [35, 36]. In addition, previous studies have suggested that participation of geriatricians in MDT discussions helps to avoid both over- and undertreatment in this subgroup of patients and the resulting complications [37, 38]. However, although the associations in our study remained significant after adjustment for some potential confounders, residual confounding cannot be excluded. Therefore, our findings do not establish that MDT attendance itself improves treatment decisions or outcomes, but rather identify MDT integration as a plausible target for future implementation and outcomes research.

Geriatric oncological surgery stands out as a particularly underdeveloped area across Europe. Despite the high risk of postoperative complications and functional decline among older adults [39], especially those living with frailty, geriatric involvement in perioperative care remains limited and often based on ad hoc consultation rather than structured co-management. This contrasts with orthogeriatric care, where co-management models are more established, and with POPS-based perioperative pathways, which emphasize early geriatric assessment, optimization, shared decision-making, prehabilitation, and coordinated postoperative care [20]. Adapting these principles to oncological surgery may be a pragmatic way to strengthen geriatric oncological surgery. The variability we report reflects the international picture described in previous literature and is further supported by emerging evidence showing that even motivated centers face significant operational and financial barriers when attempting to integrate geriatric expertise into surgical oncology workflows [16]. Strengthening perioperative geriatric collaboration models, including the establishment of co-management frameworks, should therefore be a priority for improving outcomes that matter to patients after surgery [40]. In our survey, the liaison model was the most common structure for collaborating with oncological surgery services. However, there is limited evidence on the effectiveness of liaison-based geriatric models in surgical oncology, a topic that should be addressed in future studies.

Structural implications also emerge from the persistent shortage of geriatricians, a barrier repeatedly identified in earlier and recent European studies [16, 31–33]. Expanding multidisciplinary capacity, especially through the integration of specialist nurses or trained allied health professionals in CGA workflows, represents a pragmatic strategy to broaden access while maintaining quality. This approach is consistent with successful models implemented in Belgium and other countries, where trained non-physician staff coordinate CGA and support oncologists and surgeons in decision-making [32, 33].

Finally, the inconsistent availability of national guidelines and the limited access to structured educational programs reflect the broader organizational gaps described in previous European and international studies [15, 27, 34]. Importantly, identifying these educational and structural gaps was one of the explicit aims of our survey, and our findings confirm that they remain substantial across Europe. Contradictory responses within some countries may reflect a lack of awareness or dissemination of existing initiatives, and possibly some ambiguity in our questions/options, underscoring the need for clearer visibility of national efforts and more refined data capture in future surveys. Harmonized European guidelines and structured training pathways—including dual-speciality programs and geriatric oncology and geriatric surgical oncology fellowships—could help reduce these disparities. In addition, scalable educational initiatives, such as short multidisciplinary courses, perioperative geriatric medicine masterclasses, and shared curricula for oncologists, surgeons and geriatricians, may represent pragmatic first steps, particularly in settings with limited workforce capacity. The strong clinician demand for further education highlights readiness for capacity building and suggests that implementation efforts would likely be well received.

A major strength of this study is that it presents the first European-wide survey to capture real-world geriatric oncology and geriatric oncological surgery practice across Europe. The survey achieved broad geographical coverage and provided detailed country-level analyses for nations with ten or more respondents, allowing meaningful comparisons across different healthcare systems. Its comprehensive scope, encompassing screening practices, CGA implementation, MDT participation, involvement in surgical oncology, educational structures and the availability of national guidelines, offers a valuable benchmark that clinicians and policymakers across Europe can use to assess their current practice and identify priorities for development.

Limitations of this study include the self-reported nature of the survey, which restricts the ability to infer causality and may introduce recall or reporting biases. Voluntary participation may have favored geriatricians with a particular interest in oncology and/or oncological surgery, contributing to selection bias. A major limitation is the low response rate across Europe, with many countries providing fewer than ten responses, or none at all. While the reasons for this cannot be determined, it is possible that countries with few or no participants may have limited or no structured geriatric oncology activity. This suggests that the true extent of inequality in service provision is likely to be even greater than our results indicate. Furthermore, the representativeness of country-level findings is limited. Although 11,189 emails were distributed, this denominator cannot be assumed to represent unique eligible geriatricians, because dissemination occurred through overlapping international networks and national mailing lists. Consequently, country-level results, particularly for countries with few respondents, should be interpreted as descriptive respondent-level patterns rather than nationally representative estimates of service prevalence. In addition, as center identifiers were not collected, over-representation of oncogeriatrics-interested centers cannot be excluded. Finally, although inclusion criteria were applied to ensure data quality, variable completion rates may still have led to differences in data completeness. Survey length and non-adaptive follow-up questions may have contributed to this variable completion rate.

Looking ahead, the aim should not be to define a single “best” model, but to implement the most feasible approach within local resources while safeguarding core elements that appear consistently relevant: frailty screening, GA/CGA, and opportunities for geriatrician participation into MDT decision-making—and progressively strengthening more comprehensive or co-management pathways, particularly for surgery. A European consensus on essential pathway components is needed to reduce heterogeneity, along with implementation research evaluating models such as co-management and prehabilitation, inclusion of patient-reported and caregiver outcomes, and repeated surveys with longitudinal monitoring to track progress. Addressing gaps in education, workforce capacity, guideline development, and policy support will be essential to ensure equitable and high-quality care for older adults with cancer across Europe.

In conclusion, this first European survey reveals marked heterogeneity in the organization and implementation of geriatric oncology and geriatric surgical oncology. While structured models exist in certain countries, many regions lack systematic approaches to frailty screening, CGA integration, MDT participation, and perioperative geriatric involvement. Educational gaps and inconsistent national guidance further contribute to these disparities, supporting the need for coordinated training efforts and shared minimum standards across Europe.

## Supplementary Information

Below is the link to the electronic supplementary material.

Supplementary file1 (DOCX 158 kb)

Supplementary file2 (PDF 2683 kb)

Supplementary file3 (PDF 3009 kb)

Supplementary file4 (PDF 2711 kb)

Supplementary file5 (DOCX 25 kb)

## Data Availability

The datasets generated and/or analyzed during the current study are available from the corresponding author on reasonable request.

## References

[1] Pilleron S, Sarfati D, Janssen-Heijnen M, Vignat J, Ferlay J, Bray F et al (2019) Global cancer incidence in older adults, 2012 and 2035: a population-based study. Int J Cancer 144(1):49–58. 10.1002/ijc.31664 29978474

[2] Bizuayehu HM, Ahmed KY, Kibret GD, Dadi AF, Belachew SA, Bagade T et al (2050) Global disparities of cancer and its projected burden in 2050. JAMA Netw Open 7(11):e2443198. 10.1001/jamanetworkopen.2024.43198 PMC11539015 39499513

[3] Kim DH, Rockwood K (2024) Frailty in older adults. N Engl J Med 391(6):538–548. 10.1056/NEJMra2301292 PMC11634188 39115063

[4] Burdett N, Vincent AD, O’Callaghan M, Kichenadasse G (2018) Competing risks in older patients with cancer: a systematic review of geriatric oncology trials. JNCI J Natl Cancer Inst 110(8):825–830. 10.1093/jnci/djy111 30011032

[5] Wildiers H, Heeren P, Puts M, Topinkova E, Janssen-Heijnen MLG, Extermann M et al (2014) International society of geriatric oncology consensus on geriatric assessment in older patients with cancer. J Clin Oncol 32(24):2595–2603. 10.1200/JCO.2013.54.8347 PMC4876338 25071125

[6] Dale W, Klepin HD, Williams GR, Alibhai SMH, Bergerot C, Brintzenhofeszoc K et al (2023) Practical assessment and management of vulnerabilities in older patients receiving systemic cancer therapy: ASCO guideline update. J Clin Oncol Off J Am Soc Clin Oncol 41(26):4293–4312. 10.1200/JCO.23.00933 PMC12803700 37459573

[7] Dotan E, Walter LC, Browner IS, Clifton K, Cohen HJ, Extermann M et al (2021) NCCN guidelines® insights: older adult oncology, version 1.2021: featured updates to the NCCN guidelines. J Natl Compr Canc Netw 19(9):1006–1019. 10.6004/jnccn.2021.0043 34551388

[8] Bergerot CD, Temin S, Verduzco-Aguirre HC, Aapro MS, Alibhai SMH, Aziz Z et al (2025) Geriatric assessment: ASCO global guideline. JCO Glob Oncol 11:e2500276. 10.1200/GO-25-00276 PMC13039670 40865024

[9] Cree A, Pearce J, Battisti NML, Baxter M, Brown J, Conroy S, Gomes F, Goyal N, Jones R, Lillis A, Ng C, Simcock R, Sommer D, Swinson D, Thorp N (2023) Implementing frailty assessment and management in oncology services. London: Joint Collegiate Council for Oncology; Royal College of Physicians and Royal College of Radiologists. Disponible en: https://www.rcr.ac.uk/our-services/all-our-publications/clinical-oncology-publications/implementing-frailty-assessment-and-management-in-oncology-services

[10] Parker SG, McCue P, Phelps K, McCleod A, Arora S, Nockels K et al (2018) What is comprehensive geriatric assessment (CGA)? An umbrella review. Age Ageing 47(1):149–155. 10.1093/ageing/afx166 29206906

[11] Hamaker M, Lund C, Te Molder M, Soubeyran P, Wildiers H, Van Huis L et al (2022) Geriatric assessment in the management of older patients with cancer – a systematic review (update). J Geriatr Oncol 13(6):761–777. 10.1016/j.jgo.2022.04.008 35545495

[12] Chuang MH, Chen JY, Tsai WW, Lee CW, Lee MC, Tseng WH et al (2022) Impact of comprehensive geriatric assessment on the risk of adverse events in the older patients receiving anti-cancer therapy: a systematic review and meta-analysis. Age Ageing 51(7):afac145. 10.1093/ageing/afac145 35776674

[13] Monfardini S, Perrone F, Balducci L (2023) Pitfalls in oncogeriatrics. Cancers 15(11):2910. 10.3390/cancers15112910 PMC10251873 37296871

[14] Overcash J, Cope DG, Van Cleave JH (2018) Frailty in older adults: assessment, support, and treatment implications in patients with cancer. Clin J Oncol Nurs 22(6):8–18. 10.1188/18.CJON.S2.8-18 30452021

[15] Monfardini S, Aapro MS, Bennett JM, Mori M, Regenstreif D, Rodin M et al (2007) Organization of the clinical activity of geriatric oncology: report of a SIOG (International Society of Geriatric Oncology) task force. Crit Rev Oncol Hematol 62(1):62–73. 10.1016/j.critrevonc.2006.10.003 17300950

[16] Javier-González M, Boulahssass R, Lago LD, González-Senac NM, Nair S, Vetter M (2024) Survey on current clinical practice in geriatric oncology: the individual experience in five European cancer centers. Eur Geriatr Med 16(1):125–133. 10.1007/s41999-024-01041-7 PMC11850469 39441518

[17] Chapman AE, Elias R, Plotkin E, Lowenstein LM, Swartz K (2021) Models of care in geriatric oncology. J Clin Oncol 39(19):2195–2204. 10.1200/JCO.21.00118 PMC10476747 34043453

[18] Dale W, Williams GR, R. MacKenzie A, Soto-Perez-de-Celis E, Maggiore RJ, Merrill JK et al (2021) How is geriatric assessment used in clinical practice for older adults with cancer? A survey of cancer providers by the American Society of Clinical Oncology. JCO Oncol Pract 17(6):336–344. 10.1200/OP.20.00442 PMC8462667 33064058

[19] Bréchemier D, Sourdet S, Girard P, Steinmeyer Z, Mourey L, Gérard S et al (2019) Use of comprehensive geriatric assessment (CGA) to define frailty in geriatric oncology: searching for the best threshold. Cross-sectional study of 418 old patients with cancer evaluated in the Geriatric Frailty Clinic (G.F.C.) of Toulouse (France). J Geriatr Oncol. 10(6):944–950. 10.1016/j.jgo.2019.03.011 30956134

[20] Thillainadesan J, Hilmer SN, Fleury AM, Naganathan V (2022) New horizons in the perioperative care of older adults. Age Ageing 51(2):afab245. 10.1093/ageing/afab245 35134849

[21] Van Grootven B, Mendelson DA, Deschodt M (2020) Impact of geriatric co-management programmes on outcomes in older surgical patients: update of recent evidence. Curr Opin Anaesthesiol 33(1):114–121. 10.1097/ACO.0000000000000815 31789902

[22] Joughin AL, Partridge JSL, O’Halloran T, Dhesi JK (2019) Where are we now in perioperative medicine? Results from a repeated UK survey of geriatric medicine delivered services for older people. Age Ageing 48(3):458–462. 10.1093/ageing/afy218 30624577

[23] Lodge ME, Dhesi J, Shipway DJH, Braude P, Meilak C, Partridge J et al (2024) Core elements of the perioperative medicine for older people undergoing surgery (POPS) model of care. Eur Geriatr Med 16(2):435–446. 10.1007/s41999-024-01122-7 39654009

[24] Fagard K, Deschodt M, Geyskens L, Willems S, Boland B, Wolthuis A et al (2023) Geriatric care for surgical patients: results and reflections from a cross-sectional survey in acute Belgian hospitals. Eur Geriatr Med 14(2):239–249. 10.1007/s41999-023-00748-3 PMC9870777 36690884

[25] Lodge ME, Dhesi J, Shipway DJ, Braude P, Meilak C, Partridge J et al (2024) The implementation of a perioperative medicine for older people undergoing surgery service: a qualitative case study. BMC Health Serv Res 24(1):345. 10.1186/s12913-024-10844-0 PMC10943911 38491431

[26] Marinho J, Custódio S (2022) Geriatric oncology in Portugal: where we are and what comes next—a survey of healthcare professionals. Geriatrics (Basel) 7(5):91. 10.3390/geriatrics7050091 PMC9498886 36136800

[27] Gironés R, Morilla I, Guillen-Ponce C, Torregrosa MD, Paredero I, Bustamante E et al (2018) Geriatric oncology in Spain: survey results and analysis of the current situation. Clin Transl Oncol Off Publ Fed Span Oncol Soc Natl Cancer Inst Mex 20(8):1087–1092. 10.1007/s12094-017-1813-0 PMC6061214 29327240

[28] Hauchecorne M, Pierro M, Brain E, Bringuier M, Soubeyran P, Chakiba C et al (2020) Perspectives on cancer care in older patients in France. Ecancermedicalscience 14:1103. 10.3332/ecancer.2020.1103 PMC7532034 33082853

[29] Sifer-Rivière L, Girre V, Gisselbrecht M, Saint-Jean O (2010) Physicians’ perceptions of cancer care for elderly patients: a qualitative sociological study based on a pilot geriatric oncology program. Crit Rev Oncol Hematol 75(1):58–69. 10.1016/j.critrevonc.2010.04.001 20510625

[30] Sifer-Rivière L, Saint-Jean O, Gisselbrecht M, Cudennec T, Girre V, Programme d’OncoGériatrie de l’Ouest Parisien (POGOP) (2011) What the specific tools of geriatrics and oncology can tell us about the role and status of geriatricians in a pilot geriatric oncology program. Ann Oncol Off J Eur Soc Med Oncol. 22(10):2325–2329. 10.1093/annonc/mdq748 21325447

[31] Kenis C, Heeren P, Bron D, Decoster L, Moor R, Pepersack T et al (2014) Multicenter implementation of geriatric assessment in Belgian patients with cancer: a survey on treating physicians’ general experiences and expectations. J Geriatr Oncol 5(4):431–438. 10.1016/j.jgo.2014.06.043 24986786

[32] Kenis C, Heeren P, Decoster L, Van Puyvelde K, Conings G, Cornelis F et al (2016) A Belgian survey on geriatric assessment in oncology focusing on large-scale implementation and related barriers and facilitators. J Nutr Health Aging 20(1):60–70. 10.1007/s12603-016-0677-2 PMC12878961 26728935

[33] Jonker JM, Smorenburg CH, Schiphorst AH, van Rixtel B, Portielje JEA, Hamaker ME (2014) Geriatric oncology in the Netherlands: a survey of medical oncology specialists and oncology nursing specialists. Eur J Cancer Care (Engl) 23(6):803–810. 10.1111/ecc.12193 24702775

[34] Mizutani T, Cheung KL, Hakobyan Y, Lane H, Decoster L, Karnakis T et al (2024) Leave no one behind: a global survey of the current state of geriatric oncology practice by SIOG national representatives. J Geriatr Oncol 15(2):101709. 10.1016/j.jgo.2024.101709 38310661

[35] Festen S, Kok M, Hopstaken JS, van der Wal-Huisman H, van der Leest A, Reyners AKL et al (2019) How to incorporate geriatric assessment in clinical decision-making for older patients with cancer. An implementation study. J Geriatr Oncol 10(6):951–959. 10.1016/j.jgo.2019.04.006 31031193

[36] Van Der Wal-Huisman H, Van Leeuwen BL, Stiggelbout AM, Van Nuenen FM, Van Der Weijden T, Festen S et al (2025) Integrated oncological treatment decision-making: creating a practice of patient-centred decision-making. Patient Educ Couns 131:108555. 10.1016/j.pec.2024.108555 39579519

[37] Festen S, Van Der Wal-Huisman H, Van Der Leest AHD, Reyners AKL, De Bock GH, De Graeff P et al (2021) The effect of treatment modifications by an onco-geriatric MDT on one-year mortality, days spent at home and postoperative complications. J Geriatr Oncol 12(5):779–785. 10.1016/j.jgo.2020.12.003 33342722

[38] DuMontier C, Dale W, Revette AC, Roberts J, Sanyal A, Perumal N et al (2025) Ethics of overtreatment and undertreatment in older adults with cancer. BMC Med Ethics 26(1):105. 10.1186/s12910-025-01255-9 PMC12291383 40707902

[39] Huisman MG, Ghignone F, Ugolini G, Sidorenkov G, Montroni I, Vigano A et al (2020) Long‐term survival and risk of institutionalization in onco‐geriatric surgical patients: long‐term results of the PREOP study. J Am Geriatr Soc 68(6):1235–1241. 10.1111/jgs.16384 PMC7318670 32155289

[40] Mac Eochagain C, Barrell A, Murphy J, Pattwell M, Cumming J, Slavova-Boneva V et al (2024) “What matters to you?” Patient-reported treatment goals in geriatric oncology: a cross-sectional survey. J Geriatr Oncol 15(2):101641. 10.1016/j.jgo.2023.101641 37813781

