# Supplementary material for: A survey among geriatricians on geriatric oncology and geriatric surgical oncology models in Europe
Source: Eur Geriatr Med. 2026 Aug 28;17(5):3017–36. doi: 10.1007/s41999-026-01575-y (PMC13755084; doi:10.1007/s41999-026-01575-y)
Supplement: Supplementary file 1 [file 41999_2026_1575_MOESM2_ESM.pdf]

Survey 'Oncogeriatrics in Europe: a survey among geriatricians'

Oncogeriatrics in Europe: a survey among geriatricians - Introduction

Dear colleague,

This study aims to examine how geriatric oncology is organized in Europe.

This survey is intended **exclusively for healthcare professionals specializing in geriatrics, specifically geriatricians and internists with a focus in geriatric medicine**. We kindly ask that only individuals in these specialties complete the survey.

In this study we distinguish between the **MEDICAL** geriatric oncology and the **SURGICAL** geriatric oncology. When the question concerns **SURGICAL** geriatric oncology, it will be specified.

Furthermore, the term **Comprehensive Geriatric Assessment** (CGA) is used. For this survey we focus on the CGA in the context of an oncological treatment decision. A CGA is always performed by a geriatrician (in training) or geriatric nurse (practitioner), other than the geriatric assessment (GA) which can be performed by every medical specialist or nurse (practitioner).

The results of this survey will be processed anonymously in a scientific publication and will not be traceable to the respondent. Your information will be kept confidential. **Completing the questionnaire will take about 20 minutes**. If you have any questions about the questionnaire or the study, please contact Isabelle Moens at i.moens@erasmusmc.nl

Thank you in advance for your contribution.

This survey is sent on behalf of the EuGMS SIG Gero-Oncology and SIG Perioperative Geriatric Medicine

This study was initiated by Paula Sobrini-Morillo MD, Isabelle S Moens MD, Alfonso Cruz-Jentoft MD PhD, Nicolás M. González-Senac MD, Marije Hamaker MD PhD, Francesco US Mattace-Raso MD PhD, Harmke A Polinder-Bos MD PhD

Special thanks to the following geriatricians who participated in developing this survey: Andrea Sebastiano Ciccone (France), Anja Velghe (Belgium), Cecilia Margareta Lund (Denmark), Giuseppe Dario Testa (Italy), Jugdeep Dhesi (England), Nina Neuendorff (Germany), Siri Rostoft (Norway).

Oncogeriatrics in Europe: a survey among geriatricians - Personal and work details

Number	Question	Answers
1.1	What is your profession?	☐ Geriatrician<br>☐ Internal medicine with a focus on geriatrics<br>☐ Other, please specify
1.1.1	***If 'What is your profession?' is equal to 'Other, please specify' answer this question:***<br>Please specify your profession	

1.2	How long have you been working in geriatrics?	◯ <5 years<br>◯ 5-10 years<br>◯ 11-20 years<br>◯ >20 years

1.3 In which country do you work?

- ◯ Albania
- ◯ Andorra
- ◯ Armenia
- ◯ Austria
- ◯ Belarus
- ◯ Belgium
- ◯ Bulgaria
- ◯ Croatia
- ◯ Cyprus
- ◯ Czech Republic
- ◯ Denmark
- ◯ Estonia
- ◯ Finland
- ◯ France
- ◯ Germany
- ◯ Greece
- ◯ Hungary
- ◯ Iceland
- ◯ Ireland
- ◯ Israel
- ◯ Italy
- ◯ Latvia
- ◯ Lithuania
- ◯ Luxembourg
- ◯ Malta
- ◯ Netherlands
- ◯ Northern Macedonia
- ◯ Norway
- ◯ Poland
- ◯ Portugal
- ◯ Romania
- ◯ Russia
- ◯ San Marino
- ◯ Serbia
- ◯ Slovakia
- ◯ Slovenia
- ◯ Spain
- ◯ Sweden
- ◯ Switzerland
- ◯ Turkey
- ◯ United Kingdom

1.4	In what type of hospital or clinical institution do you work?	☐ A general multi-specialty hospital<br>☐ A dedicated geriatric hospital<br>☐ A specialty cancer centre<br>☐ Outpatient only
1.5	What type of institution is your hospital?	○ University affiliated teaching hospital/ Academic Medical Centre<br>○ National health service/public hospital<br>○ Private practice/private non-teaching hospital<br>○ Other
1.5.1	***If 'What type of institution is your hospital?' is equal to 'Other' answer this question:***<br>Please specify the type of institution	
1.6	Is there a formal geriatric medicine department or section at your hospital/clinical institution?	○ Yes, a geriatric medicine department<br>○ Yes, dedicated beds for geriatric patients<br>○ No

Oncogeriatrics in Europe: a survey among geriatricians - Country details

Number	Question	Answers
2.1	Do you have a system of education and training in geriatric oncology for geriatricians (in training)?	☐ Yes, voluntary educational days for geriatricians (in training)<br>☐ Yes, obliged educational days for geriatricians (in training)<br>☐ Yes, a voluntary educational program for geriatricians (in training)<br>☐ Yes, an obliged educational program for geriatricians (in training)<br>☐ Yes, a fellowship (optional)<br>☐ Yes, a fellowship (obliged)<br>☐ No<br>☐ I don't know

2.2	Do you have any specific guidelines for treatment of older patients with cancer in your country?	◯ Yes<br>◯ Yes, for some cancer entities<br>◯ No, but there are clinical recommendations<br>◯ No<br>◯ I don't know

Oncogeriatrics in Europe: a survey among geriatricians - Current geriatric oncology practice in your institution

Number	Question	Answers
3.1	What geriatric oncology model exists in your institution?	☐ None. Patients are not referred.<br>☐ Patients are referred based upon clinical judgement of the referring physician<br>☐ Two-step model: referral to geriatrics in case of positive screening for frailty or an abnormal result according to an abbreviated GA<br>☐ A multidisciplinary consultative model: in which a multidisciplinary team assesses all patients<br>☐ There are oncologists and/or hematologists specialized in geriatrics<br>☐ Other, please specify
3.1.1	***If 'What geriatric oncology model exists in your institution?' is equal to 'Other, please specify' answer this question:***<br>Please specify what geriatric oncology models exists in your institution	
3.2	What are the referral criteria to the geriatrician?	☐ Age<br>☐ Positive screening for frailty screening test or other GA screening test<br>☐ Other, please specify
3.2.1	***If 'What are the referral criteria to the geriatrician?' is equal to 'Other, please specify' answer this question:***<br>Please specify the other referral criteria.	

3.2.2	***If 'What are the referral criteria to the geriatrician?' is equal to 'Age' answer this question:***<br>At what age are patients referred to the geriatrician?	○ 65 years and older<br>○ 70 years and older<br>○ 75 years and older<br>○ No formal age criterium is used<br>○ Other, please specify
3.2.2.1	***If 'At what age are patients referred to the geriatrician?' is equal to 'Other, please specify' answer this question:***<br>At what age are patients referred to the geriatrician, other than the above mentioned?	
3.3	Which screening scales are used to decide whether or not to refer a patient?	☐ Clinical Frailty Scale (CFS)<br>☐ Eastern Cooperative Oncology Group (ECOG) score<br>☐ Geriatric 8 (G8)<br>☐ Vulnerable Elders Survey (VES-13)<br>☐ Geriatric Assessment in Hematology (GAH)<br>☐ The Identification of Seniors At Risk-Hospitalised Patients (ISAR-HP)<br>☐ Edmonton Frail Scale (EFS)<br>☐ Groningen Frailty Indicator (GFI)<br>☐ Triage Risk Screening Tool (TRST)<br>☐ The Fried frailty phenotype (FP)<br>☐ The Barber test (BT)<br>☐ Cognitive screening 6CIT<br>☐ Cognitive screening Mini-COG<br>☐ Other, please specify
3.3.1	***If 'Which screening scales are used to decide whether or not to refer a patient?' is equal to 'Other, please specify' answer this question:***<br>Which other screening scales are used to decide whether or not to refer a patient?	
3.4	At what stage are patients with cancer usually referred to geriatricians?	☐ At diagnosis<br>☐ At start of oncological treatment<br>☐ After the start of oncological treatment<br>☐ Refractory or recurrent disease<br>☐ Once decision made for palliative treatment<br>☐ When complications/toxicity of treatment occurs<br>☐ Other, please specify

3.4.1	***If 'At what stage are patients with cancer usually referred to geriatricians?' is equal to 'Other, please specify' answer this question:***<br>At what stage, other than the above mentioned, are patients with cancer usually referred to geriatricians?	
3.5	Who performs the **comprehensive** geriatric assessment (CGA) in the geriatric oncology practice?	☐ Geriatrician (in training)<br>☐ Nurse practitioners (specialized nurses in geriatrics or geriatric oncology)<br>☐ Nurse<br>☐ Other non-physician specialties, e.g., physiotherapist, gerontologist, social worker, psychologist, occupational therapist<br>☐ Other, please specify
3.5.1	***If 'Who performs the comprehensive geriatric assessment (CGA) in the geriatric oncology practice?' is equal to 'Other, please specify' answer this question:***<br>Who, other than the above mentioned, performs the comprehensive geriatric assessment (CGA) in the geriatric oncology practice?	
3.6	In the situation of performing a **comprehensive** geriatric assessment (CGA), how much time does the physician have available per patient?	○ 30 minutes<br>○ 60 minutes<br>○ 90 minutes<br>○ Depends on the situation, no standard time<br>○ Not applicable (we do not perform CGA in our hospital)<br>○ Other, please specify
3.6.1	***If 'In the situation of performing a comprehensive geriatric assessment (CGA), how much time does the physician have available per patient?' is equal to 'Other, please specify' answer this question:***<br>In the situation of performing a comprehensive geriatric assessment (CGA), how much time does the physician have available per patient, other than the before mentioned times?	
3.7	In the case of a geriatric assessment, (**GA**), how much time does the physician have available per patient?	○ <15 minutes<br>○ 15-30 minutes<br>○ Not applicable (we do not perform GA in our hospital)<br>○ I do not know, because it is only performed by other medical specialists<br>○ Other, please specify

3.7.1	***If 'In the case of a geriatric assessment, (GA), how much time does the physician have available per patient?' is equal to 'Other, please specify' answer this question:***<br>In the case of a geriatric assessment, (GA), how much time is available per patient, other than the above mentioned answers?	
3.8	Based on your **comprehensive** geriatric assessment, do you only give a conclusion of the geriatric domains and possible impairments, or do you also provide treatment recommendations?	○ Only conclusion<br>○ Conclusions and treatment recommendations only for the oncological treatment<br>○ Conclusions and treatment recommendations only for optimisation of CGA domains<br>○ Conclusions and treatment recommendations for optimisation of CGA domains and for oncological treatment
3.9	How are **comprehensive** geriatric assessment conclusions and recommendations shared with the oncologist or other referring specialist?	☐ Reported in a multidisciplinary team meeting<br>☐ By phone<br>☐ A written summary in the patient file<br>☐ Other, please specify
3.9.1	***If 'How are comprehensive geriatric assessment conclusions and recommendations shared with the oncologist or other referring specialist?' is equal to 'Other, please specify' answer this question:***<br>How are comprehensive geriatric assessment conclusions and recommendations shared with the oncologist or other referring specialist, other than the before mentioned answer options?	
3.10	Do you follow-up patients during and/or after the oncological treatment period?	☐ Yes, routinely for all<br>☐ Yes, but only for research<br>☐ On request only, in case of complications or a defined need<br>☐ No
3.11	If you routinely follow up patients, at what time interval from first consult is this provided?	○ After 3 months<br>○ After 6 months<br>○ After 9 months<br>○ Not applicable<br>○ Other, please specify
3.11.1	***If 'If you routinely follow up patients, at what time interval from first consult is this provided?' is equal to 'Other, please specify' answer this question:***<br>If you routinely follow up patients, at what time interval from first consult is this provided, other than the above mentioned times?	

3.12	If you routinely follow up patients, who performs this?	☐ Geriatrician (or geriatrician in training)<br>☐ Nurse practitioners (specialized nurses in geriatrics or geriatric oncology)<br>☐ Not applicable<br>☐ Other, please specify
3.12.1	***If 'If you routinely follow up patients, who performs this?' is equal to 'Other, please specify' answer this question:***<br>If you routinely follow up patients, who, other than the geriatrician (in training) or nurse practitioner performs this?	
3.13	If you routinely provide follow up to patients, how is this delivered?	☐ Face-to-face consultation<br>☐ Telephone consultation<br>☐ Not applicable<br>☐ Other, please specify
3.13.1	***If 'If you routinely provide follow up to patients, how is this delivered?' is equal to 'Other, please specify' answer this question:***<br>If you routinely provide follow up to patients, how is this delivered, other than the before mentionend?	
3.14	Do you take part in multidisciplinary oncological meetings?	☐ Yes, in casual (incidental) meetings<br>☐ Yes, routinely in tumour-specific meetings<br>☐ Yes, routinely in oncogeriatric meetings (not tumour specific)<br>☐ Yes, routinely in pre-surgery meetings (not tumour specific)<br>☐ No, someone else of the geriatric team (e.g. nurse) participates<br>☐ No, none of the members of the geriatric team participates

3.15	If you take part in multidisciplinary tumour-specific oncological meetings, which meetings (tumour types) do you attend?	☐ Bladder cancer<br>☐ Breast cancer<br>☐ Colorectal cancer<br>☐ Head and neck cancer<br>☐ Hematological cancer<br>☐ Hepato-biliary cancer<br>☐ Kidney (renal cell) cancer<br>☐ Lung cancer<br>☐ Neuro-oncology<br>☐ Orthopedic oncology<br>☐ Pancreatic cancer<br>☐ Prostate cancer<br>☐ Skin cancer<br>☐ Upper GI-tract cancer<br>☐ Gynecological cancer<br>☐ Other, please specify<br>☐ Not applicable
3.15.1	***If 'If you take part in multidisciplinary tumour-specific oncological meetings, which meetings (tumour types) do you attend?' is equal to 'Other, please specify' answer this question:***<br>If you take part in multidisciplinary tumour-specific oncological meetings: which meetings (tumour types) do you attend, other than the above mentioned?	
3.16	Do you actively participate in the treatment decision-making process?	○ Yes<br>○ In some cases<br>○ No
3.17	Do you get positive feedback from the oncological/surgical care-provider that they appreciate your geriatric input?	○ Yes<br>○ Sometimes<br>○ No
3.18	Do you offer a pre-habilitation program at your institution (e.g. one or more interventions of exercise, nutrition, psychological strategies that aims to enhance functional capacity)?	○ Yes<br>○ No<br>○ I don't know
3.18.1	***If 'Do you offer a pre-habilitation program at your institution (e.g. one or more interventions of exercise, nutrition, psychological strategies that aims to enhance functional capacity)?' is equal to 'Yes' answer this question:***<br>What does the pre-habilitation program consist of?	☐ Exercise<br>☐ Nutrition<br>☐ Psychological<br>☐ Quit smoking/alcohol program<br>☐ Other, please specify

3.18.1.1	***If 'What does the pre-habilitation program consist of?' is equal to 'Other, please specify' answer this question:*** What does your pre-habilitation program consist of, other than the above mentioned?	

Oncogeriatrics in Europe: a survey among geriatricians - Co-management for hospitalized SURGICAL oncology patients

Number	Question	Answers
4.1	Are you involved in the management of patients during their hospitalization for oncological surgery?	○ Yes<br>○ No
4.2	Do you have a structural form of collaboration with oncological surgery?	○ Yes, a liaison model (the geriatrician is asked for advice and/or co-management for specific patients with e.g. a delirium, or for patients who received a pre-surgery CGA)<br>○ Yes, a co-management model (the geriatrician is structurally present at the surgical ward for advise and/or co-management)<br>○ Yes, other please specify<br>○ No
4.2.1	***If 'Do you have a structural form of collaboration with oncological surgery?' is equal to 'Yes, other please specify' answer this question:*** Please specify the structural form of collaboration with oncological surgery	

4.3	In case you are involved in the management of patients during their hospitalization for oncological surgery, for which tumor types?	☐ Bladder cancer<br>☐ Breast cancer<br>☐ Colorectal cancer<br>☐ Head and neck cancer<br>☐ Hematological cancer<br>☐ Hepato-biliary cancer<br>☐ Kidney (renal cell) cancer<br>☐ Lung cancer<br>☐ Neuro-oncology<br>☐ Orthopedic oncology<br>☐ Pancreatic cancer<br>☐ Prostate cancer<br>☐ Skin cancer<br>☐ Upper GI-tract cancer<br>☐ Gynecological cancer<br>☐ Other, please specify<br>☐ Not applicable
4.3.1	***If 'In case you are involved in the management of patients during their hospitalization for oncological surgery, for which tumor types?' is equal to 'Other, please specify' answer this question:***<br>In case you are involved in the management of patients during their hospitalization for oncological surgery, for which tumor types other than the above mentioned?	
4.4	How many patients do you treat (in liaison or structural co-management) during their hospitalization for oncological surgery?	○ <5 new patients/week<br>○ 5-10 new patients/week<br>○ >10 new patients/week<br>○ Not applicable

Oncogeriatrics in Europe: a survey among geriatricians - Barriers and improvements for geriatric oncology in your institution

Number	Question	Answers

5.1	What do you think are the main limitations for the development of geriatric *MEDICAL* oncology in your country?	☐ Lack of awareness and interest among geriatricians<br>☐ Lack of awareness and interest among other medical specialists (e.g. oncologist, surgeons)<br>☐ Lack of awareness and interest among policy makers<br>☐ Lack of evidence<br>☐ Lack of time<br>☐ Lack of support staff<br>☐ Lack of funding/reimbursement<br>☐ Lack of education on geriatric oncology<br>☐ Lack of communication between involved healthcare professionals<br>☐ Lack of geriatricians<br>☐ Other, please specify
5.1.1	***If 'What do you think are the main limitations for the development of geriatric MEDICAL oncology in your country?' is equal to 'Other, please specify' answer this question:***<br>Please specify other limitations for the development of geriatric *MEDICAL* oncology in your country.	
5.2	What do you think are the main limitations for the development of geriatric *SURGICAL* oncology in your country?	☐ Lack of awareness and interest among geriatricians<br>☐ Lack of awareness and interest among other medical specialists (e.g. oncologist, surgeons)<br>☐ Lack of awareness and interest among policy makers<br>☐ Lack of evidence<br>☐ Lack of time<br>☐ Lack of support staff<br>☐ Lack of funding/reimbursement<br>☐ Lack of education on geriatric oncology<br>☐ Lack of communication between involved healthcare professionals<br>☐ Lack of geriatricians<br>☐ Other, please specify
5.2.1	***If 'What do you think are the main limitations for the development of geriatric SURGICAL oncology in your country?' is equal to 'Other, please specify' answer this question:***<br>Please specify other limitations for the development of geriatric *SURGICAL* oncology in your country.	

5.3	What solutions or actions do you envision to promote the development of geriatric *MEDICAL* oncology (oncology, hematology, radiotherapy) in your country?	☐ Education for geriatricians<br>☐ Education for oncologists, haematologists, radiotherapists<br>☐ Implementation of dual-training programs or dedicated geriatric oncology fellowships<br>☐ Influence on political stake-holders (e.g. by European medical societies)<br>☐ Regulatory solutions (e.g. obligatory use of (C)GA at certified cancer centres)<br>☐ Other, please specify
5.3.1	***If 'What solutions or actions do you envision to promote the development of geriatric MEDICAL oncology (oncology, hematology, radiotherapy) in your country?' is equal to 'Other, please specify' answer this question:***<br>Please specify other solutions or actions do you envision to promote the development of geriatric *MEDICAL* oncology (oncology, hematology, radiotherapy) in your country.	
5.4	What solutions or actions do you envision to promote the development of geriatric *SURGICAL* oncology in your country?	☐ Education for geriatricians<br>☐ Education for surgical specialists<br>☐ Implementation of dual-training programs or dedicated geriatric oncology fellowships<br>☐ Influence on political stake-holders (e.g. by European medical societies)<br>☐ Regulatory solutions (e.g. obligatory use of (C)GA at certified cancer centres)<br>☐ Other, please specify
5.4.1	***If 'What solutions or actions do you envision to promote the development of geriatric SURGICAL oncology in your country?' is equal to 'Other, please specify' answer this question:***<br>Please specify other solutions or actions do you envision to promote the development of geriatric *SURGICAL* oncology in your country.	
5.4.2	***If 'What solutions or actions do you envision to promote the development of geriatric SURGICAL oncology in your country?' is equal to 'Education for geriatricians' answer this question:***<br>What areas of perioperative geriatric medicine would you like to learn more about?	
5.5	Would you be interested in a masterclass on perioperative geriatric medicine?	○ Yes<br>○ No<br>○ I don't know

Oncogeriatrics in Europe: a survey among geriatricians - Outro

To complete the survey, please click “finish survey” at the bottom of this page. Thank you for your participation. If you have any additional comments/suggestions, please e-mail Isabelle Moens (i.moens@erasmusmc.nl).
