# Supplementary material for: A survey among geriatricians on geriatric oncology and geriatric surgical oncology models in Europe
Source: Eur Geriatr Med. 2026 Aug 28;17(5):3017–36. doi: 10.1007/s41999-026-01575-y (PMC13755084; doi:10.1007/s41999-026-01575-y)
Supplement: Supplementary file 2 [file 41999_2026_1575_MOESM3_ESM.pdf]

Survey 'Oncogériatrie en Europe: une enquête auprès des gériatres'

Oncogériatrie en Europe: une enquête auprès des gériatres - Introduction

Cher collègue,

Cette étude vise à examiner comment l'oncologie gériatrique est organisée en Europe.

Cette enquête est destinée **exclusivement aux professionnels de santé spécialisés en gériatrie, notamment les gériatres et les internistes ayant une spécialisation en médecine gériatrique**. Nous vous prions de bien vouloir remplir ce sondage uniquement si vous exercez dans ces spécialités.

Dans cette étude, nous distinguons entre l'oncologie gériatrique **MÉDICALE** et l'oncologie gériatrique **CHIRURGICALE**. Lorsque la question concerne l'oncologie gériatrique **CHIRURGICALE**, cela sera spécifié.

De plus, le terme **Comprehensive Geriatric Assessment** (CGA) est utilisé. Pour cette enquête, nous nous concentrons sur le CGA dans le cadre d'une décision de traitement oncologique. Un CGA est toujours réalisé par un gériatre (en formation) ou une infirmière gériatrique (praticienne), contrairement à Geritric Assessment (GA) qui peut être réalisée par tout spécialiste médical ou infirmière (praticienne).

Les résultats de cette enquête seront traités anonymement dans une publication scientifique et ne seront pas traçables au répondant. Vos informations resteront confidentielles. **Remplir le questionnaire prendra environ 20 minutes**. Si vous avez des questions concernant le questionnaire ou l'étude, veuillez contacter Isabelle Moens à i.moens@erasmusmc.nl

Merci d'avance pour votre contribution!

Cette enquête est envoyée au nom du SIG Géro-Oncologie de l'EuGMS et du SIG Médecine Gériatrique Périopératoire de l'EuGMS.

Cette étude a été initiée par Paula Sobrini-Morillo MD, Isabelle S Moens MD, Alfonso Cruz-Jentoft MD PhD, Nicolás M. González-Senac MD, Marije Hamaker MD PhD, Francesco US Mattace-Raso MD PhD, Harmke A Polinder-Bos MD PhD

Remerciements particuliers aux gériatres suivants qui ont participé à l'élaboration de cette enquête : Andrea Sebastiano Ciccone (France), Anja Velghe (Belgium), Cecilia Margareta Lund (Denmark), Giuseppe Dario Testa (Italy), Jugdeep Dhesi (England), Nina Neuendorff (Germany), Siri Rostoft (Norway).

Oncogériatrie en Europe: une enquête auprès des gériatres - Détails personnels et professionnels

Number	Question	Answers
1.1	Quelle est votre profession?	☐ Gériatre<br>☐ Médecine interne avec une spécialisation en gériatrie<br>☐ Autre, veuillez préciser

1.1.1	***If 'Quelle est votre profession?' is equal to 'Autre, veuillez préciser' answer this question:***<br>Veuillez spécifier votre profession	
1.2	Depuis combien de temps travaillez-vous en gériatrie ?	○ <5 ans<br>○ 5-10 ans<br>○ 11-20 ans<br>○ > 20 ans

1.3 Dans quel pays travaillez-vous ?

- ◯ Albania
- ◯ Andorra
- ◯ Armenia
- ◯ Austria
- ◯ Belarus
- ◯ Belgium
- ◯ Bulgaria
- ◯ Croatia
- ◯ Cyprus
- ◯ Czech Republic
- ◯ Denmark
- ◯ Estonia
- ◯ Finland
- ◯ France
- ◯ Germany
- ◯ Greece
- ◯ Hungary
- ◯ Iceland
- ◯ Ireland
- ◯ Israel
- ◯ Italy
- ◯ Latvia
- ◯ Lithuania
- ◯ Luxembourg
- ◯ Malta
- ◯ Netherlands
- ◯ Northern Macedonia
- ◯ Norway
- ◯ Poland
- ◯ Portugal
- ◯ Romania
- ◯ Russia
- ◯ San Marino
- ◯ Serbia
- ◯ Slovakia
- ◯ Slovenia
- ◯ Spain
- ◯ Sweden
- ◯ Switzerland
- ◯ Turkey
- ◯ United Kingdom

1.4	Dans quel type d'hôpital ou d'établissement clinique travaillez-vous ?	☐ Un hôpital général à spécialités multiples<br>☐ Un hôpital gériatrique dédié<br>☐ Un centre spécialisé en oncologie<br>☐ Uniquement en consultation externe
1.5	Quel type d'établissement est votre hôpital ?	○ Hôpital universitaire/ Centre médical académique<br>○ Hôpital public/ Service de santé national<br>○ Cabinet privé/hôpital privé non universitaire<br>○ Autre
1.5.1	***If 'Quel type d'établissement est votre hôpital ?' is equal to 'Autre' answer this question:***<br>Veuillez préciser le type d'établissement	
1.6	Y a-t-il un département ou une section formelle de médecine gériatrique dans votre hôpital/établissement clinique ?	○ Oui, un département de médecine gériatrique<br>○ Oui, des lits dédiés aux patients gériatriques<br>○ Non

Oncogériatrie en Europe: une enquête auprès des gériatres - Détails du pays

Number	Question	Answers
2.1	Avez-vous un système d'éducation et de formation en oncologie gériatrique pour les gériatres (en formation)?	☐ Oui, des journées éducatives volontaires pour les gériatres (en formation)<br>☐ Oui, des journées éducatives obligatoires pour les gériatres (en formation)<br>☐ Oui, un programme éducatif volontaire pour les gériatres (en formation)<br>☐ Oui, un programme éducatif obligatoire pour les gériatres (en formation)<br>☐ Oui, une bourse (optionnelle)<br>☐ Oui, une bourse (obligatoire)<br>☐ Non<br>☐ Je ne sais pas

2.2	Avez-vous des directives spécifiques pour le traitement des patients âgés atteints de cancer dans votre pays?	◯ Oui<br>◯ Oui, pour certaines entités cancéreuses<br>◯ Non, mais il existe des recommandations cliniques<br>◯ Non<br>◯ Je ne sais pas

Oncogériatrie en Europe: une enquête auprès des gériatres - Pratique actuelle de l'oncologie gériatrique dans votre établissement

Number	Question	Answers
3.1	Quel modèle d'oncologie gériatrique existe dans votre institution?	☐ Aucun. Les patients ne sont pas référés.<br>☐ Les patients sont référés en fonction du jugement clinique du médecin référant<br>☐ Modèle en deux étapes: référer à la gériatrie en cas de dépistage positif de la fragilité ou d'un résultat anormal selon un GA abrégé<br>☐ Modèle consultatif pluridisciplinaire : une équipe pluridisciplinaire évalue tous les patients<br>☐ Il y a des oncologues et/ou des hématologues spécialisés en gériatrie<br>☐ Autre, veuillez préciser
3.1.1	***If 'Quel modèle d'oncologie gériatrique existe dans votre institution?' is equal to 'Autre, veuillez préciser' answer this question:***<br>Veuillez préciser quels modèles d'oncologie gériatrique existent dans votre institution	
3.2	Quels sont les critères d'orientation vers le gériatre ?	☐ Âge<br>☐ Dépistage positif du test de dépistage de la fragilité ou d'un autre test de dépistage GA<br>☐ Autre, veuillez préciser
3.2.1	***If 'Quels sont les critères d'orientation vers le gériatre ?' is equal to 'Autre, veuillez préciser' answer this question:***<br>Veuillez préciser les autres critères d'orientation	

3.2.2	***If 'Quels sont les critères d'orientation vers le gériatre ?' is equal to 'Âge' answer this question:*** À quel âge les patients sont-ils orientés vers le gériatre?	◯ 65 ans et plus<br>◯ 70 ans et plus<br>◯ 75 ans et plus<br>◯ Aucun critère d'âge formel n'est utilisé<br>◯ Autre, veuillez préciser
3.2.2.1	***If 'À quel âge les patients sont-ils orientés vers le gériatre?' is equal to 'Autre, veuillez préciser' answer this question:*** À quel âge les patients sont-ils orientés vers le gériatre, autres que ceux mentionnés ci-dessus?	
3.3	Quelles échelles de dépistage sont utilisées pour décider de référer ou non un patient?	☐ Clinical Frailty Scale (CFS)<br>☐ Eastern Cooperative Oncology Group (ECOG) score<br>☐ Geriatric 8 (G8)<br>☐ Vulnerable Elders Survey (VES-13)<br>☐ Geriatric Assessment in Hematology (GAH)<br>☐ The Identification of Seniors At Risk-Hospitalised Patients (ISAR-HP)<br>☐ Edmonton Frail Scale (EFS)<br>☐ Groningen Frailty Indicator (GFI)<br>☐ Triage Risk Screening Tool (TRST)<br>☐ The Fried frailty phenotype (FP)<br>☐ The Barber test (BT)<br>☐ Cognitive screening 6CIT<br>☐ Cognitive screening Mini-COG<br>☐ Other, please specify
3.3.1	***If 'Quelles échelles de dépistage sont utilisées pour décider de référer ou non un patient?' is equal to 'Other, please specify' answer this question:*** Quelles autres échelles de dépistage sont utilisées pour décider de référer ou non un patient?	
3.4	À quel stade les patients atteints de cancer sont-ils habituellement orientés vers les gériatres?	☐ Au diagnostic<br>☐ Au début du traitement oncologique<br>☐ Après le début du traitement oncologique<br>☐ Maladie réfractaire ou récidivante<br>☐ Une fois la décision prise pour un traitement palliatif<br>☐ Lorsque des complications/toxicités du traitement surviennent<br>☐ Autre, veuillez préciser

3.4.1	***If 'À quel stade les patients atteints de cancer sont-ils habituellement orientés vers les gériatres?' is equal to 'Autre, veuillez préciser' answer this question:***<br>À quel stade, autre que ceux mentionnés ci-dessus, les patients atteints de cancer sont-ils habituellement orientés vers les gériatres?	
3.5	Qui réalise the **comprehensive** geriatric assessment (CGA) dans la pratique d'oncologie gériatrique?	☐ Gériatre (en formation)<br>☐ Infirmières praticiennes (infirmières spécialisées en gériatrie ou oncologie gériatrique)<br>☐ Infirmière<br>☐ Autres spécialités non-médicales, par exemple, gérontologue, physiothérapeute, assistant social, psychologue, ergothérapeute<br>☐ Autre, veuillez préciser
3.5.1	***If 'Qui réalise the comprehensive geriatric assessment (CGA) dans la pratique d'oncologie gériatrique?' is equal to 'Autre, veuillez préciser' answer this question:***<br>Qui, autre que ceux mentionnés ci-dessus, réalise the comprehensive geriatric assessment (CGA) dans la pratique d'oncologie gériatrique ?	
3.6	Dans le cas de la réalisation d'une **comprehensive** geriatric assessment (CGA), combien de temps le médecin a-t-il par patient?	○ 30 minutes<br>○ 60 minutes<br>○ 90 minutes<br>○ Cela dépend de la situation, aucun temps standard<br>○ Non applicable (nous ne réalisons pas de CGA dans notre hôpital)<br>○ Autre, veuillez préciser
3.6.1	***If 'Dans le cas de la réalisation d'une comprehensive geriatric assessment (CGA), combien de temps le médecin a-t-il par patient?' is equal to 'Autre, veuillez préciser' answer this question:***<br>Dans le cas de la réalisation d'une comprehensive geriatric assessment (CGA), combien de temps le médecin a-t-il par patient, autre que les temps mentionnés ci-dessus?	
3.7	Dans le cas d'une geriatric assessment, (**GA**), combien de temps le médecin a-t-il par patient?	○ Moins de 15 minutes<br>○ 15-30 minutes<br>○ Non applicable (nous ne réalisons pas de GA dans notre hôpital)<br>○ Je ne sais pas, car ce n'est réalisé que par d'autres spécialistes médicaux<br>○ Autre, veuillez préciser

3.7.1	***If 'Dans le cas d'une geriatric assessment, (GA), combien de temps le médecin a-t-il par patient?' is equal to 'Autre, veuillez préciser' answer this question:***<br>Dans le cas d'une geriatric assessment, (GA), , combien de temps est disponible par patient, autre que les réponses mentionnées ci-dessus?	
3.8	Sur la base de votre **comprehensive** geriatric assessment, fournissez-vous uniquement une conclusion sur les domaines gériatriques et les dysfonctionnements possibles, ou proposez-vous également des recommandations de traitement?	○ Seulement une conclusion<br>○ Conclusions et recommandations de traitement uniquement pour le traitement oncologique<br>○ Conclusions et recommandations de traitement uniquement pour l'optimisation des domaines de comprehensive geriatric assesment (CGA)<br>○ Conclusions et recommandations de traitement pour l'optimisation des domaines de comprehensive geriatric assesment (CGA) et pour le traitement oncologique
3.9	Comment les conclusions et recommandations de **comprehensive** geriatric assessment sont-elles partagées avec l'oncologue ou autre spécialiste référent?	☐ Rapportées lors d'une réunion d'équipe pluridisciplinaire<br>☐ Par téléphone<br>☐ Un résumé écrit dans le dossier du patient<br>☐ Autre, veuillez préciser
3.9.1	***If 'Comment les conclusions et recommandations de comprehensive geriatric assessment sont-elles partagées avec l'oncologue ou autre spécialiste référent?' is equal to 'Autre, veuillez préciser' answer this question:***<br>Comment les conclusions et recommandations de l'évaluation gériatrique complète sont-elles partagées avec l'oncologue ou autre spécialiste référent, autres que les options de réponse mentionnées ci-dessus?	
3.10	Assurez-vous un suivi des patients pendant et/ou après la période de traitement oncologique ?	☐ Oui, systématiquement pour tous<br>☐ Oui, mais uniquement pour la recherche<br>☐ Uniquement sur demande, en cas de complications ou de besoin défini<br>☐ Non
3.11	Si vous suivez systématiquement les patients, à quel intervalle de temps depuis la première consultation cela est-il effectué?	○ Après 3 mois<br>○ Après 6 mois<br>○ Après 9 mois<br>○ Non applicable<br>○ Autre, veuillez préciser

3.11.1	***If 'Si vous suivez systématiquement les patients, à quel intervalle de temps depuis la première consultation cela est-il effectué?' is equal to 'Autre, veuillez préciser' answer this question:***<br>Si vous suivez systématiquement les patients, à quel intervalle de temps depuis la première consultation cela est-il effectué, autre que les temps mentionnés ci-dessus?	
3.12	Si vous suivez systématiquement les patients, qui effectue ce suivi?	☐ Gériatre (ou gériatre en formation)<br>☐ Infirmières praticiennes (infirmières spécialisées en gériatrie ou oncologie gériatrique)<br>☐ Non applicable<br>☐ Autre, veuillez préciser
3.12.1	***If 'Si vous suivez systématiquement les patients, qui effectue ce suivi?' is equal to 'Autre, veuillez préciser' answer this question:***<br>Si vous suivez systématiquement les patients, qui, autre que le gériatre (en formation) ou l'infirmière praticienne effectue ce suivi?	
3.13	Si vous suivez systématiquement les patients, comment ce suivi est-il réalisé?	☐ Consultation en face à face<br>☐ Consultation téléphonique<br>☐ Non applicable<br>☐ Autre, veuillez préciser
3.13.1	***If 'Si vous suivez systématiquement les patients, comment ce suivi est-il réalisé?' is equal to 'Autre, veuillez préciser' answer this question:***<br>Si vous suivez systématiquement les patients, comment ce suivi est-il réalisé, autre que les réponses mentionnées ci-dessus?	
3.14	Participez-vous à des réunions oncologiques pluridisciplinaires?	☐ Oui, lors de réunions occasionnelles (incidentes)<br>☐ Oui, systématiquement lors de réunions spécifiques aux tumeurs<br>☐ Oui, systématiquement lors de réunions oncogériatriques (non spécifiques aux tumeurs)<br>☐ Oui, systématiquement lors de réunions pré-chirurgicales (non spécifiques aux tumeurs)<br>☐ Non, un autre membre de l'équipe gériatrique (par exemple, infirmière) participe<br>☐ Non, aucun membre de l'équipe gériatrique ne participe

3.15	Si vous participez à des réunions oncologiques pluridisciplinaires spécifiques aux tumeurs, à quelles réunions (types de tumeurs) assistez-vous ?	☐ Cancer de la vessie<br>☐ Cancer du sein<br>☐ Cancer colorectal<br>☐ Cancer de la tête et du cou<br>☐ Cancer hématologique<br>☐ Cancer hépatobiliaire<br>☐ Cancer du rein (carcinome rénal)<br>☐ Cancer du poumon<br>☐ Neuro-oncologie<br>☐ Oncologie orthopédique<br>☐ Cancer du pancréas<br>☐ Cancer de la prostate<br>☐ Cancer de la peau<br>☐ Cancer du tractus gastro-intestinal supérieur<br>☐ Cancer gynécologique<br>☐ Autre, veuillez préciser<br>☐ Non applicable
3.15.1	***If 'Si vous participez à des réunions oncologiques pluridisciplinaires spécifiques aux tumeurs, à quelles réunions (types de tumeurs) assistez-vous ?' is equal to 'Autre, veuillez préciser' answer this question:***<br>Si vous participez à des réunions oncologiques pluridisciplinaires spécifiques aux tumeurs : à quelles réunions (types de tumeurs) assistez-vous, autres que celles mentionnées ci-dessus?	
3.16	Participez-vous activement au processus de prise de décision thérapeutique?	○ Oui<br>○ Dans certains cas<br>○ Non
3.17	Recevez-vous des retours positifs de la part du prestataire de soins oncologiques/chirurgicaux indiquant qu'il apprécie votre apport gériatrique?	○ Oui<br>○ Parfois<br>○ Non
3.18	Proposez-vous un programme de pré-habilitation dans votre établissement (par exemple, une ou plusieurs interventions d'exercice, de nutrition, de stratégies psychologiques visant à améliorer la capacité fonctionnelle)?	○ Oui<br>○ Non<br>○ Je ne sais pas

3.18.1	***If 'Proposez-vous un programme de pré-habilitation dans votre établissement (par exemple, une ou plusieurs interventions d'exercice, de nutrition, de stratégies psychologiques visant à améliorer la capacité fonctionnelle)?' is equal to 'Oui' answer this question:*** En quoi consiste le programme de pré-habilitation?	☐ Exercice<br>☐ Nutrition<br>☐ Psychologique<br>☐ Programme pour arrêter de fumer/boire de l'alcool<br>☐ Autre, veuillez préciser
3.18.1.1	***If 'En quoi consiste le programme de pré-habilitation?' is equal to 'Autre, veuillez préciser' answer this question:*** En quoi consiste votre programme de pré-habilitation, autre que les éléments mentionnés ci-dessus?	

Oncogériatrie en Europe: une enquête auprès des gériatres - Co-gestion des patients CHIRURGICAUX oncologiques hospitalisés

Number	Question	Answers
4.1	Êtes-vous impliqué dans la gestion des patients pendant leur hospitalisation pour une chirurgie oncologique?	◯ Oui<br>◯ Non
4.2	Avez-vous une forme de collaboration structurée avec la chirurgie oncologique?	◯ Oui, un modèle de liaison (le gériatre est sollicité pour des conseils et/ou une co-gestion pour des patients spécifiques avec, par exemple, un délirium, ou pour les patients ayant reçu une évaluation gériatrique globale préopératoire)<br>◯ Oui, un modèle de co-gestion (le gériatre est structurellement présent dans le service de chirurgie pour des conseils et/ou une co-gestion)<br>◯ Oui, autre, veuillez préciser<br>◯ Non
4.2.1	***If 'Avez-vous une forme de collaboration structurée avec la chirurgie oncologique?' is equal to 'Oui, autre, veuillez préciser' answer this question:*** Veuillez préciser la forme de collaboration structurée avec la chirurgie oncologique	

4.3	Si vous êtes impliqué dans la gestion des patients pendant leur hospitalisation pour une chirurgie oncologique, pour quels types de tumeurs?	☐ Cancer de la vessie<br>☐ Cancer du sein<br>☐ Cancer colorectal<br>☐ Cancer de la tête et du cou<br>☐ Cancer hématologique<br>☐ Cancer hépatobiliaire<br>☐ Cancer du rein (carcinome rénal)<br>☐ Cancer du poumon<br>☐ Neuro-oncologie<br>☐ Oncologie orthopédique<br>☐ Cancer du pancréas<br>☐ Cancer de la prostate<br>☐ Cancer de la peau<br>☐ Cancer du tractus gastro-intestinal supérieur<br>☐ Cancer gynécologique<br>☐ Autre, veuillez préciser<br>☐ Non applicable
4.3.1	***If 'Si vous êtes impliqué dans la gestion des patients pendant leur hospitalisation pour une chirurgie oncologique, pour quels types de tumeurs?' is equal to 'Autre, veuillez préciser' answer this question:***<br>Si vous êtes impliqué dans la gestion des patients pendant leur hospitalisation pour une chirurgie oncologique, pour quels types de tumeurs autres que ceux mentionnés ci-dessus?	
4.4	Combien de patients traitez-vous (en liaison ou en co-gestion structurée) pendant leur hospitalisation pour une chirurgie oncologique?	○ Moins de 5 nouveaux patients/semaine<br>○ 5-10 nouveaux patients/semaine<br>○ Plus de 10 nouveaux patients/semaine<br>○ Non applicable

Oncogériatrie en Europe: une enquête auprès des gériatres - Obstacles et améliorations pour l'oncologie gériatrique dans votre établissement

Number	Question	Answers

5.1	Quels sont, selon vous, les principaux obstacles au développement de l'oncologie gériatrique *MÉDICALE* dans votre pays?	☐ Manque de sensibilisation et d'intérêt parmi les gériatres<br>☐ Manque de sensibilisation et d'intérêt parmi les autres spécialistes médicaux (par exemple, oncologues, chirurgiens)<br>☐ Manque de sensibilisation et d'intérêt parmi les décideurs politiques<br>☐ Manque de preuves<br>☐ Manque de temps<br>☐ Manque de personnel de soutien<br>☐ Manque de financement/remboursement<br>☐ Manque de formation en oncologie gériatrique<br>☐ Manque de communication entre les professionnels de santé impliqués<br>☐ Manque de gériatres<br>☐ Autre, veuillez préciser
5.1.1	***If 'Quels sont, selon vous, les principaux obstacles au développement de l'oncologie gériatrique MÉDICALE dans votre pays?' is equal to 'Autre, veuillez préciser' answer this question:***<br>Veuillez préciser d'autres obstacles au développement de l'oncologie gériatrique *MÉDICALE* dans votre pays.	
5.2	Quels sont, selon vous, les principaux obstacles au développement de l'oncologie gériatrique *CHIRURGICALE* dans votre pays?	☐ Manque de sensibilisation et d'intérêt parmi les gériatres<br>☐ Manque de sensibilisation et d'intérêt parmi les autres spécialistes médicaux (par exemple, oncologues, chirurgiens)<br>☐ Manque de sensibilisation et d'intérêt parmi les décideurs politiques<br>☐ Manque de preuves<br>☐ Manque de temps<br>☐ Manque de personnel de soutien<br>☐ Manque de financement/remboursement<br>☐ Manque de formation en oncologie gériatrique<br>☐ Manque de communication entre les professionnels de santé impliqués<br>☐ Manque de gériatres<br>☐ Autre, veuillez préciser
5.2.1	***If 'Quels sont, selon vous, les principaux obstacles au développement de l'oncologie gériatrique CHIRURGICALE dans votre pays?' is equal to 'Autre, veuillez préciser' answer this question:***<br>Veuillez préciser d'autres obstacles au développement de l'oncologie gériatrique *CHIRURGICALE* dans votre pays.	

5.3	Quelles solutions ou actions envisagez-vous pour promouvoir le développement de l'oncologie gériatrique *MÉDICALE* (oncologie, hématologie, radiothérapie) dans votre pays?	☐ Formation pour les gériatres<br>☐ Formation pour les oncologues, hématologues, radiothérapeutes<br>☐ Mise en œuvre de programmes de double formation ou de fellowships dédiés à l'oncologie gériatrique<br>☐ Influence sur les parties prenantes politiques (par exemple, par les sociétés médicales européennes)<br>☐ Solutions réglementaires (par exemple, utilisation obligatoire du (C)GA dans les centres de cancer certifiés)<br>☐ Autre, veuillez préciser
5.3.1	***If 'Quelles solutions ou actions envisagez-vous pour promouvoir le développement de l'oncologie gériatrique MÉDICALE (oncologie, hématologie, radiothérapie) dans votre pays?' is equal to 'Autre, veuillez préciser' answer this question:***<br>Veuillez préciser d'autres solutions ou actions que vous envisagez pour promouvoir le développement de l'oncologie gériatrique *MÉDICALE* (oncologie, hématologie, radiothérapie) dans votre pays.	
5.4	Quelles solutions ou actions envisagez-vous pour promouvoir le développement de l'oncologie gériatrique *CHIRURGICALE* dans votre pays ?	☐ Formation pour les gériatres<br>☐ Formation pour les spécialistes chirurgicaux<br>☐ Mise en œuvre de programmes de double formation ou de fellowships dédiés à l'oncologie gériatrique<br>☐ Influence sur les parties prenantes politiques (par exemple, par les sociétés médicales européennes)<br>☐ Solutions réglementaires (par exemple, utilisation obligatoire du (C)GA dans les centres de cancer certifiés)<br>☐ Autre, veuillez préciser
5.4.1	***If 'Quelles solutions ou actions envisagez-vous pour promouvoir le développement de l'oncologie gériatrique CHIRURGICALE dans votre pays ?' is equal to 'Autre, veuillez préciser' answer this question:***<br>Veuillez préciser d'autres solutions ou actions que vous envisagez pour promouvoir le développement de l'oncologie gériatrique *CHIRURGICALE* dans votre pays.	
5.4.2	***If 'Quelles solutions ou actions envisagez-vous pour promouvoir le développement de l'oncologie gériatrique CHIRURGICALE dans votre pays ?' is equal to 'Formation pour les gériatres' answer this question:***<br>Dans quels domaines de la médecine gériatrique périopératoire aimeriez-vous en savoir plus?	

5.5	Seriez-vous intéressé(e) par un masterclass sur la médecine gériatrique périopératoire?	○ Oui<br>○ Non<br>○ Je ne sais pas

Oncogériatrie en Europe: une enquête auprès des gériatres - Outro

Pour compléter l'enquête, veuillez cliquer sur "terminer l'enquête / finish survey" en bas de cette page. Merci pour votre participation. Si vous avez des commentaires/suggestions supplémentaires, veuillez envoyer un e-mail à Isabelle Moens (i.moens@erasmusmc.nl).
