# Supplementary material for: A survey among geriatricians on geriatric oncology and geriatric surgical oncology models in Europe
Source: Eur Geriatr Med. 2026 Aug 28;17(5):3017–36. doi: 10.1007/s41999-026-01575-y (PMC13755084; doi:10.1007/s41999-026-01575-y)
Supplement: Supplementary file 3 [file 41999_2026_1575_MOESM4_ESM.pdf]

Survey 'Avrupa Ülkelerinde Geriatrik Onkoloji: Geriatristler için Anket Çalışması'

Avrupa Ülkelerinde Geriatrik Onkoloji: Geriatristler için Anket Çalışması - Introduction

Değerli meslektaşımız,

Bu çalışma, Avrupa'da geriatrik onkoloji alanının nasıl organize edildiğini incelemeyi amaçlamaktadır.

Bu anket **yalnızca geriyatri konusunda uzmanlaşmış sağlık profesyonelleri için, özellikle geriyatristler ve geriyatri tıbbına odaklanan iç hastalıkları uzmanları** için hazırlanmıştır. Sadece bu uzmanlık alanlarında çalışan kişilerin anketi doldurmasını rica ederiz.

Bu çalışmada, **MEDIKAL** geriatrik onkoloji ve **CERRAHI** geriatrik onkoloji ayrı ayrı incelenmektedir. Size yöneltilen soru **CERRAHI** geriatrik onkoloji ile ilgili olduğunda, bu özellikle belirtilmiştir.

Ayrıca, bu ankette "Kapsamlı Geriatrik Değerlendirme" (KGD) terimi sıklıkla kullanılmaktadır. Bu anket için, KGD'ye onkolojik tedavi kararı bağlamında odaklanıyoruz. Bu çalışmada KGD, yalnızca bir geriatri uzmanı (veya uzmanlık eğitimi alan bir geriatri asistanı) ya da geriatri hemşiresi tarafından gerçekleştirilen, onkolojik bir tedavi kararı bağlamında odaklanılan bir değerlendirmeyi ifade etmektedir. KGD dışında, geriatrik değerlendirme (GD) ise her tıbbi uzman veya hemşire tarafından yapılabilir.

Bu anketin sonuçları bilimsel bir yayında anonim olarak işlenecek ve katılımcılarla ilişkilendirilemeyecektir. Bilgileriniz gizli tutulacaktır. **Anketi doldurmak yaklaşık 20 dakika sürecektir**. Anket veya çalışma hakkında herhangi bir sorunuz varsa, lütfen Isabelle Moens ile i.moens@erasmusmc.nl adresinden iletişime geçin.

Katkılarınız için şimdiden teşekkür ederiz.

Bu anket, EuGMS SIG Gero-Onkoloji ve SIG Perioperatif Geriatrik Tıp adına gönderilmektedir.

Bu çalışma, aşağıdaki uzmanlar tarafından başlatılmıştır: Paula Sobrini-Morillo MD, Isabelle S Moens MD, Alfonso Cruz-Jentoft MD PhD, Nicolás M. González-Senac MD, Marije Hamaker MD PhD, Francesco US Mattace-Raso MD PhD, Harmke A Polinder-Bos MD PhD.

Anketin geliştirilmesine katkıda bulunan aşağıdaki geriatristlere özel teşekkürler: Andrea Sebastiano Ciccone (Fransa), Anja Velghe (Belçika), Cecilia Margareta Lund (Danimarka), Giuseppe Dario Testa (İtalya), Jugdeep Dhesi (İngiltere), Nina Neuendorff (Almanya), Siri Rostoft (Norveç).

Avrupa Ülkelerinde Geriatrik Onkoloji: Geriatristler için Anket Çalışması - Kişisel Bilgiler ve Çalışma Bilgileri

Number	Question	Answers
1.1	Mesleğiniz nedir?	☐ Geriatrist<br>☐ İç Hastalıkları, Geriatriye odaklanmış<br>☐ Diğer, lütfen belirtin

1.1.1	***If 'Mesleğiniz nedir?' is equal to 'Diğer, lütfen belirtin' answer this question:***<br>Lütfen mesleğinizi belirtiniz	
1.2	Ne kadar zamandır geriyatri alanında çalışıyorsunuz?	◯ <5 yıl<br>◯ 5-10 yıl<br>◯ 11-20 yıl<br>◯ >20 yıl

1.3 Hangi ülkede çalışıyorsunuz?

- ◯ Arnavutluk
- ◯ Andorra
- ◯ Ermenistan
- ◯ Avusturya
- ◯ Belarus
- ◯ Belçika
- ◯ Bulgaristan
- ◯ Hırvatistan
- ◯ Kıbrıs
- ◯ Çek Cumhuriyeti
- ◯ Danimarka
- ◯ Estonya
- ◯ Finlandiya
- ◯ Fransa
- ◯ Almanya
- ◯ Yunanistan
- ◯ Macaristan
- ◯ İzlanda
- ◯ İrlanda
- ◯ İsrail
- ◯ İtalya
- ◯ Letonya
- ◯ Litvanya
- ◯ Lüksemburg
- ◯ Malta
- ◯ Hollanda
- ◯ Kuzey Makedonya
- ◯ Norveç
- ◯ Polonya
- ◯ Portekiz
- ◯ Romanya
- ◯ Rusya
- ◯ San Marino
- ◯ Sırbistan
- ◯ Slovakya
- ◯ Slovenya
- ◯ İspanya
- ◯ İsveç
- ◯ İsviçre
- ◯ Türkiye
- ◯ Birleşik Krallık

1.4	Hangi tür bir hastane veya klinik kurumunda çalışıyorsunuz?	☐ Genel, çok branşlı hastane<br>☐ Yaşlı bakımına özel bir hastane<br>☐ Spesifik Kanser merkezi (Hastanesi)<br>☐ Sadece poliklinik hizmeti veren bir kurum
1.5	Hastaneniz hangi tür bir kuruma aittir?	○ Üniversiteye bağlı bir eğitim hastanesi / Akademik Tıp Merkezi<br>○ Devlet/ kamu hastanesi<br>○ Özel bir muayenehane / Özel bir akademik olmayan hastane<br>○ Diğer
1.5.1	***If 'Hastaneniz hangi tür bir kuruma aittir?' is equal to 'Diğer' answer this question:***<br>Lütfen kurumun türünü belirtiniz.	
1.6	Hastanenizde/klinik kurumunuzda resmi bir geriatri departmanı veya bölümü var mı?	○ Evet, bir geriatri departmanı var<br>○ Evet, yaşlı hastalara ayrılmış yataklar mevcut<br>○ Hayır

Avrupa Ülkelerinde Geriatrik Onkoloji: Geriatristler için Anket Çalışması - Ülke Bilgileri

Number	Question	Answers
2.1	Geriatri uzmanları (veya uzmanlık eğitimi alanlar) için geriatrik onkoloji alanında bir eğitim ve öğretim sisteminiz var mı?	☐ Evet, geriatri uzmanları (veya uzmanlık eğitimi alanlar) için gönüllü eğitim günleri<br>☐ Evet, geriatri uzmanları (veya uzmanlık eğitimi alanlar) için zorunlu eğitim günleri<br>☐ Evet, geriatri uzmanları (veya uzmanlık eğitimi alanlar) için gönüllü bir eğitim programı<br>☐ Evet, geriatri uzmanları (veya uzmanlık eğitimi alanlar) için zorunlu bir eğitim programı<br>☐ Evet, tercihe bağlı bir burs programı<br>☐ Evet, zorunlu bir burs programı<br>☐ Hayır<br>☐ Bilmiyorum

2.2	Ülkenizde yaşlı kanser hastalarının tedavisi için belirli kılavuzlar var mı?	○ Evet<br>○ Evet, bazı kanser türleri için<br>○ Hayır, ancak klinik öneriler mevcut<br>○ Hayır<br>○ Bilmiyorum

Avrupa Ülkelerinde Geriatrik Onkoloji: Geriatristler için Anket Çalışması - Kurumunuzda mevcut geriatrik onkoloji uygulamaları

Number	Question	Answers
3.1	Kurumunuzda hangi geriatrik onkoloji modeli bulunmaktadır?	☐ Hiçbiri. Hastalar yönlendirilmemektedir.<br>☐ Hastalar, ilgili hekimin klinik değerlendirmesine göre yönlendirilmektedir.<br>☐ İki aşamalı model: Kırılganlık için pozitif tarama sonucu ya da kısa geriatrik değerlendirmeye göre anormal bir sonuç durumunda geriatriye yönlendirme.<br>☐ Multidisipliner danışma modeli: Multidisipliner bir ekibin tüm hastaları değerlendirdiği model.<br>☐ Geriatri konusunda uzmanlaşmış onkologlar ve/veya hematologlar bulunmaktadır.<br>☐ Diğer, lütfen belirtiniz
3.1.1	***If 'Kurumunuzda hangi geriatrik onkoloji modeli bulunmaktadır?' is equal to 'Diğer, lütfen belirtiniz' answer this question:***<br>Lütfen kurumunuzda hangi geriatrik onkoloji modellerinin bulunduğunu belirtiniz.	
3.2	Hastaların geriatri uzmanına yönlendirilme kriterleri nelerdir?	☐ Yaş<br>☐ Kırılganlık tarama testi veya diğer GD tarama testleri için pozitif sonuç<br>☐ Diğer, lütfen belirtin
3.2.1	***If 'Hastaların geriatri uzmanına yönlendirilme kriterleri nelerdir?' is equal to 'Diğer, lütfen belirtin' answer this question:***<br>Lütfen diğer yönlendirme kriterlerini belirtiniz.	

3.2.2	***If 'Hastaların geriatri uzmanına yönlendirilme kriterleri nelerdir?' is equal to 'Yaş' answer this question:***<br>Hastalar hangi yaşta geriatri uzmanına yönlendirilmektedir?	◯ 65 yaş ve üzeri<br>◯ 70 yaş ve üzeri<br>◯ 75 yaş ve üzeri<br>◯ Resmi bir yaş kriteri kullanılmamaktadır<br>◯ Diğer, lütfen belirtiniz
3.2.2.1	***If 'Hastalar hangi yaşta geriatri uzmanına yönlendirilmektedir?' is equal to 'Diğer, lütfen belirtiniz' answer this question:***<br>Hastalar yukarıda belirtilenlerin dışında hangi yaşta geriatri uzmanına yönlendirilmektedir?	
3.3	Bir hastayı yönlendirip yönlendirmemeye karar vermek için hangi tarama ölçekleri kullanılmaktadır?	☐ Clinical Frailty Scale (CFS)<br>☐ Eastern Cooperative Oncology Group (ECOG) score<br>☐ Geriatric 8 (G8)<br>☐ Vulnerable Elders Survey (VES-13)<br>☐ Geriatric Assessment in Hematology (GAH)<br>☐ The Identification of Seniors At Risk-Hospitalised Patients (ISAR-HP)<br>☐ Edmonton Frail Scale (EFS)<br>☐ Groningen Frailty Indicator (GFI)<br>☐ Triage Risk Screening Tool (TRST)<br>☐ The Fried frailty phenotype (FP)<br>☐ The Barber test (BT)<br>☐ Cognitive screening 6CIT<br>☐ Cognitive screening Mini-COG<br>☐ Diğer, lütfen belirtiniz
3.3.1	***If 'Bir hastayı yönlendirip yönlendirmemeye karar vermek için hangi tarama ölçekleri kullanılmaktadır?' is equal to 'Diğer, lütfen belirtiniz' answer this question:***<br>Hangi diğer tarama ölçekleri bir hastayı yönlendirip yönlendirmeme kararını vermek için kullanılır?	
3.4	Kanser hastaları genellikle hangi aşamada geriatristlere yönlendirilir?	☐ Tanı sırasında<br>☐ Onkolojik tedavinin başlangıcında<br>☐ Onkolojik tedavi başladıktan sonra<br>☐ Refrakter veya tekrarlayan hastalık durumunda<br>☐ Palyatif tedavi kararı alındığında<br>☐ Tedavinin komplikasyonları/toksisitesi ortaya çıktığında<br>☐ Diğer, lütfen belirtiniz

3.4.1	***If 'Kanser hastaları genellikle hangi aşamada geriatristlere yönlendirilir?' is equal to 'Diğer, lütfen belirtiniz' answer this question:***<br>Yukarıda belirtilenlerin dışında, kanser hastaları genellikle hangi aşamada geriatristlere yönlendirilir?	
3.5	Geriatrik onkoloji pratiğinde **kapsamlı** geriatrik değerlendirmeyi (KGD) kim gerçekleştirir?	☐ Geriatrist (eğitim sürecinde)<br>☐ Spesifik Hemşire (geriatri veya geriatrik onkoloji konusunda uzmanlaşmış hemşireler)<br>☐ Hemşire<br>☐ Diğer hekim olmayan uzmanlık alanları, örneğin gerontolog, fizyoterapist, sosyal hizmet uzmanı, psikolog, meslek terapisti<br>☐ Diğer, lütfen belirtiniz
3.5.1	***If 'Geriatrik onkoloji pratiğinde kapsamlı geriatrik değerlendirmeyi (KGD) kim gerçekleştirir?' is equal to 'Diğer, lütfen belirtiniz' answer this question:***<br>Yukarıda belirtilenlerin dışında, geriatrik onkoloji pratiğinde kapsamlı geriatrik değerlendirmeyi (KGD) kim gerçekleştirir?	
3.6	**Kapsamlı** geriatrik değerlendirme (KGD) yapma durumunda, hekim hasta başına ne kadar zaman ayırabiliyor?	○ 30 dakika<br>○ 60 dakika<br>○ 90 dakika<br>○ Duruma bağlı, standart bir süre yok<br>○ Uygulanamaz (hastanemizde KGD yapılmıyor)<br>○ Diğer, lütfen belirtiniz
3.6.1	***If 'Kapsamlı geriatrik değerlendirme (KGD) yapma durumunda, hekim hasta başına ne kadar zaman ayırabiliyor?' is equal to 'Diğer, lütfen belirtiniz' answer this question:***<br>Kapsamlı geriatrik değerlendirme (KGD) yapma durumunda, yukarıda belirtilen süreler dışında, hekim hasta başına ne kadar zaman ayırabiliyor?	
3.7	3.7 Geriatrik değerlendirme (**GD**) durumunda, hekim hasta başına ne kadar zaman ayırabiliyor?	○ <15 dakika<br>○ 15-30 dakika<br>○ Uygulanamaz (hastanemizde GD yapılmıyor)<br>○ Bilmiyorum, çünkü yalnızca diğer tıbbi uzmanlar tarafından yapılıyor<br>○ Diğer, lütfen belirtiniz

3.7.1	***If '3.7 Geriatrik değerlendirme (GD) durumunda, hekim hasta başına ne kadar zaman ayırabiliyor?' is equal to 'Diğer, lütfen belirtiniz' answer this question:***<br>Geriatrik değerlendirme (GD) durumunda, yukarıda belirtilen cevaplar dışında, hasta başına ne kadar zaman ayrılabiliyor?	
3.8	**Kapsamlı** geriatrik değerlendirmenize dayanarak, sadece geriatrik alanlar ve olası bozukluklar hakkında bir sonuç mu veriyorsunuz, yoksa tedavi önerileri de sunuyor musunuz?	◯ Sadece sonuç veriyorum<br>◯ Sadece onkolojik tedavi için sonuçlar ve tedavi önerileri veriyorum<br>◯ Sadece KGD’deki sorunların düzeltilmesi için sonuçlar ve tedavi önerileri veriyorum<br>◯ KGD’deki sorunların düzeltilmesi ve onkolojik tedavi için sonuçlar ve tedavi önerileri veriyorum
3.9	**Kapsamlı** geriatrik değerlendirme sonuçları ve önerileri onkolog veya diğer yönlendiren uzmanla nasıl paylaşılır?	☐ Multidisipliner bir ekip toplantısında raporlanır<br>☐ Telefonla<br>☐ Hasta dosyasına yazılı bir özet olarak eklenir<br>☐ Diğer, lütfen belirtiniz
3.9.1	***If 'Kapsamlı geriatrik değerlendirme sonuçları ve önerileri onkolog veya diğer yönlendiren uzmanla nasıl paylaşılır?' is equal to 'Diğer, lütfen belirtiniz' answer this question:***<br>Kapsamlı geriatrik değerlendirme sonuçları ve önerileri, yukarıda belirtilen cevap seçenekleri dışında, onkolog veya diğer yönlendiren uzmanla nasıl paylaşılır?	
3.10	Onkolojik tedavi süreci sırasında ve/veya sonrasında hastaları takip ediyor musunuz?	☐ Evet, rutin olarak tüm hastalar için<br>☐ Evet, ancak sadece araştırma amacıyla<br>☐ Sadece talep üzerine, komplikasyonlar veya belirli bir ihtiyaç durumunda<br>☐ Hayır
3.11	Eğer hastaları rutin olarak takip ediyorsanız, bu takip ilk konsültasyondan itibaren hangi zaman aralığında yapılır?	◯ 3 ay sonra<br>◯ 6 ay sonra<br>◯ 7 ay sonra<br>◯ Uygulanamaz<br>◯ Diğer, lütfen belirtiniz
3.11.1	***If 'Eğer hastaları rutin olarak takip ediyorsanız, bu takip ilk konsültasyondan itibaren hangi zaman aralığında yapılır?' is equal to 'Diğer, lütfen belirtiniz' answer this question:***<br>Eğer hastaları rutin olarak takip ediyorsanız, yukarıda belirtilen süreler dışında, bu takip ilk konsültasyondan itibaren hangi zaman aralığında yapılır?	

3.12	Eğer hastaları rutin olarak takip ediyorsanız, bu takibi kim gerçekleştirir?	☐ Geriatrist (veya eğitim aşamasında olan geriatrist)<br>☐ Spesifik Hemşire (geriatri veya geriatrik onkoloji konusunda uzmanlaşmış hemşireler)<br>☐ Uygulanamaz<br>☐ Diğer, lütfen belirtiniz
3.12.1	***If 'Eğer hastaları rutin olarak takip ediyorsanız, bu takibi kim gerçekleştirir?' is equal to 'Diğer, lütfen belirtiniz' answer this question:***<br>Eğer hastaları rutin olarak takip ediyorsanız, bunu geriatri uzmanı (eğitimde olan) veya pratisyen hemşire dışında kim yapıyor?	
3.13	Eğer hastalara rutin olarak takip sağlıyorsanız, bu takip nasıl yapılmaktadır?	☐ Yüz yüze görüşme<br>☐ Telefon görüşmesi<br>☐ Uygulanamaz<br>☐ Diğer, lütfen belirtiniz
3.13.1	***If 'Eğer hastalara rutin olarak takip sağlıyorsanız, bu takip nasıl yapılmaktadır?' is equal to 'Diğer, lütfen belirtiniz' answer this question:***<br>Eğer hastalara rutin olarak takip sağlıyorsanız, bu daha önce belirtilen yöntemler dışında nasıl yapılmaktadır?	
3.14	Multidisipliner onkolojik toplantılara katılıyor musunuz?	☐ Evet, gayriresmî (tesadüfî) toplantılarda<br>☐ Evet, rutin olarak tümör spesifik toplantılarda<br>☐ Evet, rutin olarak onkogeriyatrik toplantılarda (tümör spesifik olmayan)<br>☐ Evet, rutin olarak ameliyat öncesi toplantılarda (tümör spesifik olmayan)<br>☐ Hayır, geriatri ekibinden başka biri (ör. hemşire) katılıyor<br>☐ Hayır, geriatri ekibinin hiçbir üyesi katılmıyor

3.15	Eğer multidisipliner tümör-spesifik onkolojik toplantılara katılıyorsanız, hangi toplantılara (tümör türleri) katılıyorsunuz?	☐ Mesane kanseri<br>☐ Meme kanseri<br>☐ Kolorektal kanser<br>☐ Baş ve boyun kanseri<br>☐ Hematolojik kanser<br>☐ Hepato-biliyer kanser<br>☐ Böbrek (renal hücreli) kanseri<br>☐ Akciğer kanseri<br>☐ Nöro-onkoloji<br>☐ Ortopedik onkoloji<br>☐ Pankreas kanseri<br>☐ Prostat kanseri<br>☐ Cilt kanseri<br>☐ Üst gastrointestinal sistem kanseri<br>☐ Jinekolojik kanser<br>☐ Diğer, lütfen belirtiniz<br>☐ Uygulanamaz
3.15.1	***If 'Eğer multidisipliner tümör-spesifik onkolojik toplantılara katılıyorsanız, hangi toplantılara (tümör türleri) katılıyorsunuz?' is equal to 'Diğer, lütfen belirtiniz' answer this question:***<br>Eğer multidisipliner tümör-spesifik onkolojik toplantılara katılıyorsanız: yukarıda belirtilenlerin dışında hangi toplantılara (tümör türleri) katılıyorsunuz?	
3.16	Tedavi karar verme sürecine aktif olarak katılıyor musunuz?	○ Evet<br>○ Bazı durumlarda<br>○ Hayır
3.17	Onkolojik/cerrahi hekimlerinden, geriatrik katkınızı takdir ettiklerine dair olumlu geri bildirim alıyor musunuz?	○ Evet<br>○ Bazen<br>○ Hayir
3.18	Kurumunuzda bir ön rehabilitasyon programı sunuyor musunuz (örneğin, fonksiyonel kapasiteyi artırmayı hedefleyen egzersiz, beslenme, psikolojik destek stratejilerinden biri veya daha fazlası)?	○ Evet<br>○ Hayir<br>○ Bilmiyorum
3.18.1	***If 'Kurumunuzda bir ön rehabilitasyon programı sunuyor musunuz (örneğin, fonksiyonel kapasiteyi artırmayı hedefleyen egzersiz, beslenme, psikolojik destek stratejilerinden biri veya daha fazlası)?' is equal to 'Evet' answer this question:***<br>Kurumunuzdaki ön rehabilitasyon programı nelerden oluşmaktadır?	☐ Egzersiz<br>☐ Beslenme<br>☐ Psikolojik<br>☐ Sigara/alkol bırakma programı<br>☐ Diğer, lütfen belirtiniz

3.18.1.1	***If 'Kurumunuzdaki ön rehabilitasyon programı nelerden oluşmaktadır?' is equal to 'Diğer, lütfen belirtiniz' answer this question:*** Kurumunuzdaki ön rehabilitasyon programı, yukarıda belirtilenlerin dışında nelerden oluşmaktadır?	

Avrupa Ülkelerinde Geriatrik Onkoloji: Geriatristler için Anket Çalışması - Hastanede yatan CERRAHI onkoloji hastaları için eş yönetim

Number	Question	Answers
4.1	Onkolojik cerrahi sırasında hastaların yönetiminde yer alıyor musunuz?	◯ Evet<br>◯ Hayir
4.2	Onkolojik cerrahi ile yapısal bir iş birliği modeliniz var mı?	◯ Evet, bir irtibat modeli (Geriatri uzmanından, örneğin deliryum gelişen veya ameliyat öncesi KGD yapılan hastalar için öneri ve/veya eş yönetim istenir)<br>◯ Evet, bir eş yönetim modeli (geriatri uzmanı, cerrahi serviste öneri ve/veya eş yönetim için yapısal olarak bulunur)<br>◯ Evet, diğer (lütfen belirtiniz)<br>◯ Hayır
4.2.1	***If 'Onkolojik cerrahi ile yapısal bir iş birliği modeliniz var mı?' is equal to 'Evet, diğer (lütfen belirtiniz)' answer this question:*** Lütfen onkolojik cerrahi ile olan yapısal iş birliği modelini belirtiniz.	

4.3	Eğer onkolojik cerrahi için yatan hastaların yönetiminde yer alıyorsanız, hangi tümör türleri için katılım sağlıyorsunuz?	☐ Mesane kanseri<br>☐ Meme kanseri<br>☐ Kolorektal kanser<br>☐ Baş ve boyun kanseri<br>☐ Hematolojik kanser<br>☐ Hepato-biliyer kanser<br>☐ Böbrek (renal hücreli) kanseri<br>☐ Akciğer kanseri<br>☐ Nöro-onkoloji<br>☐ Ortopedik onkoloji<br>☐ Pankreas kanseri<br>☐ Prostat kanseri<br>☐ Cilt kanseri<br>☐ Üst gastrointestinal sistem kanseri<br>☐ Jinekolojik kanser<br>☐ Diğer, lütfen belirtiniz<br>☐ Uygulanamaz
4.3.1	***If 'Eğer onkolojik cerrahi için yatan hastaların yönetiminde yer alıyorsanız, hangi tümör türleri için katılım sağlıyorsunuz?' is equal to 'Diğer, lütfen belirtiniz' answer this question:***<br>Eğer onkolojik cerrahi sırasında hastaların yönetiminde yer alıyorsanız, yukarıda belirtilenlerin dışında hangi tümör türleri için katılım sağlıyorsunuz?	
4.4	Onkolojik cerrahi nedeniyle hastanede yatan hastaların tedavisinde (irtibat modeli veya yapısal eş yönetim kapsamında) kaç hasta tedavi ediyorsunuz?	○ Haftada <5 yeni hasta<br>○ Haftada 5-10 yeni hasta<br>○ Haftada >10 yeni hasta<br>○ Uygulanamaz

Avrupa Ülkelerinde Geriatrik Onkoloji: Geriatristler için Anket Çalışması - Kurumunuzda Geriatrik Onkoloji İçin Engeller ve İyileştirme Önerileri

Number	Question	Answers

5.1	Sizce ülkenizde geriatrik *MEDIKAL* onkoloji alanının gelişimindeki başlıca sınırlamalar nelerdir?	☐ Geriatristler arasında farkındalık ve ilgi eksikliği<br>☐ Diğer tıbbi uzmanlar (ör. onkologlar, cerrahlar) arasında farkındalık ve ilgi eksikliği<br>☐ Politika yapıcılar arasında farkındalık ve ilgi eksikliği<br>☐ Kanıt eksikliği<br>☐ Zaman eksikliği<br>☐ Destek personeli eksikliği<br>☐ Finansman/geri ödeme eksikliği<br>☐ Geriatrik onkoloji konusunda eğitim eksikliği<br>☐ İlgili sağlık profesyonelleri arasında iletişim eksikliği<br>☐ Geriatrist eksikliği<br>☐ Diğer, lütfen belirtiniz
5.1.1	***If 'Sizce ülkenizde geriatrik MEDIKAL onkoloji alanının gelişimindeki başlıca sınırlamalar nelerdir?' is equal to 'Diğer, lütfen belirtiniz' answer this question:***<br>Lütfen ülkenizde geriatrik *MEDIKAL* onkoloji alanının gelişimindeki diğer sınırlamaları belirtiniz.	
5.2	Ülkenizde geriatrik *CERRAHI* onkoloji alanının gelişimindeki başlıca sınırlamalar nelerdir	☐ Geriatristler arasında farkındalık ve ilgi eksikliği<br>☐ Diğer tıbbi uzmanlar (ör. onkologlar, cerrahlar) arasında farkındalık ve ilgi eksikliği<br>☐ Politika yapıcılar arasında farkındalık ve ilgi eksikliği<br>☐ Kanıt eksikliği<br>☐ Zaman eksikliği<br>☐ Destek personeli eksikliği<br>☐ Finansman/geri ödeme eksikliği<br>☐ Geriatrik onkoloji konusunda eğitim eksikliği<br>☐ İlgili sağlık profesyonelleri arasında iletişim eksikliği<br>☐ Geriatrist eksikliği<br>☐ Diğer, lütfen belirtiniz
5.2.1	***If 'Ülkenizde geriatrik CERRAHI onkoloji alanının gelişimindeki başlıca sınırlamalar nelerdir' is equal to 'Diğer, lütfen belirtiniz' answer this question:***<br>Lütfen ülkenizde geriatrik *CERRAHI* onkolojinin gelişimi için diğer kısıtlamaları belirtiniz	

5.3	Ülkenizde geriatrik *MEDIKAL* onkoloji (onkoloji, hematoloji, radyasyon onkolojisi) alanının gelişimini teşvik etmek için hangi çözümleri veya eylemleri öngörüyorsunuz?	☐ Geriatristler için eğitim<br>☐ Onkologlar, hematologlar ve radyasyon onkologları için eğitim<br>☐ Çift eğitim programlarının uygulanması veya geriatrik onkolojiye özel yan dal uzmanlık programlarının geliştirilmesi<br>☐ Siyasi paydaşlar üzerinde etki yaratmak (ör. Avrupa tıp dernekleri aracılığıyla)<br>☐ Düzenleyici çözümler (ör. sertifikalı kanser merkezlerinde KGD kullanımının zorunlu hale getirilmesi<br>☐ Diğer, lütfen belirtiniz
5.3.1	***If 'Ülkenizde geriatrik MEDIKAL onkoloji (onkoloji, hematoloji, radyasyon onkolojisi) alanının gelişimini teşvik etmek için hangi çözümleri veya eylemleri öngörüyorsunuz?' is equal to 'Diğer, lütfen belirtiniz' answer this question:***<br>Lütfen ülkenizde geriatrik *MEDIKAL* onkoloji (onkoloji, hematoloji, radyasyon onkolojisi) alanının gelişimini teşvik etmek için öngördüğünüz diğer çözümleri veya eylemleri belirtiniz.	
5.4	Ülkenizde geriatrik *CERRAHI* onkoloji alanının gelişimini teşvik etmek için hangi çözümleri veya eylemleri öngörüyorsunuz?	☐ Geriatristler için eğitim<br>☐ Cerrahi uzmanları için eğitim<br>☐ Çift eğitim programlarının uygulanması veya geriatrik onkolojiye özel yan dal uzmanlık programlarının geliştirilmesi<br>☐ Siyasi paydaşlar üzerinde etki yaratmak (ör. Avrupa tıp dernekleri aracılığıyla)<br>☐ Düzenleyici çözümler (ör. sertifikalı kanser merkezlerinde KGD kullanımının zorunlu hale getirilmesi)<br>☐ Diğer, lütfen belirtiniz
5.4.1	***If 'Ülkenizde geriatrik CERRAHI onkoloji alanının gelişimini teşvik etmek için hangi çözümleri veya eylemleri öngörüyorsunuz?' is equal to 'Diğer, lütfen belirtiniz' answer this question:***<br>Lütfen ülkenizde geriatrik *CERRAHI* onkoloji (onkoloji, hematoloji, radyoterapi) alanının gelişimini teşvik etmek için öngördüğünüz diğer çözümleri veya eylemleri belirtiniz.	
5.4.2	***If 'Ülkenizde geriatrik CERRAHI onkoloji alanının gelişimini teşvik etmek için hangi çözümleri veya eylemleri öngörüyorsunuz?' is equal to 'Geriatristler için eğitim' answer this question:***<br>Perioperatif geriyatri tıbbının hangi alanları hakkında daha fazla bilgi edinmek istersiniz?	

5.5	Perioperatif geriyatri tıbbı üzerine bir masterclass'a ilgi duyar mısınız?	◯ Evet<br>◯ Hayir<br>◯ Bilmiyorum

Avrupa Ülkelerinde Geriatrik Onkoloji: Geriatristler için Anket Çalışması - Outro

Anketi tamamlamak için lütfen bu sayfanın altındaki "anketi bitir / finish survey" butonuna tıklayın. Katılımınız için teşekkür ederiz. Eklemek istediğiniz yorumlar veya önerileriniz varsa, lütfen Isabelle Moens'e (i.moens@erasmusmc.nl) e-posta gönderin.
