# Supplementary material for: A survey among geriatricians on geriatric oncology and geriatric surgical oncology models in Europe
Source: Eur Geriatr Med. 2026 Aug 28;17(5):3017–36. doi: 10.1007/s41999-026-01575-y (PMC13755084; doi:10.1007/s41999-026-01575-y)
Supplement: Supplementary file 4 [file 41999_2026_1575_MOESM1_ESM.docx]

**Supplementary file**

**Fig. S1** Reponses by country

Figure S1. Number and percentage of responses by country, grouped by European region. Horizontal bars display the total number of survey responses for each country; labels at the end of each bar indicate *n* (%) of the overall sample. Countries are color-coded by region as defined below.

Eastern Europe: Belarus, Bulgaria, Hungary, Poland, Romania, Slovakia, Turkey.

Western Europe: Austria, Belgium, France, Germany, Luxemburg, the Netherlands, Switzerland.

Northern Europe: Denmark, Estonia, Finland, Iceland, Ireland, Lithuania, Norway, Sweden, United Kingdom.

Southern Europe: Albania, Andorra, Croatia, Greece, Israel, Italy, Malta, Portugal, Serbia, Slovenia, Spain.

**Summary of the respondents, geriatricians or internal medicine physicians specialising in geriatrics, with a survey response rate of at least 26%, from the 24 countries not included in the in-depth analysis.**

Albania

*n* = 1 respondent (median survey progress: 38%).

The respondent was a physician specialising in internal medicine and geriatrics, working in a specialist cancer centre. The respondent reported that there is no oncogeriatric education or training system in the country, nor any available guidelines. The respondent (R) also reported that there is no formal oncogeriatric model in place, and that patients are not referred to geriatrics/oncogeriatrics. Nevertheless, R would be interested in attending a masterclass on perioperative geriatric medicine. The remaining information is either missing or unspecified.

Andorra

*n* = 2 respondents (median survey progress: 100 %)

The respondents were geriatricians working in general, multi-specialty hospitals. They reported that there is no oncogeriatric education or training system in their country, nor any available guidelines. They also stated that there is no formal oncogeriatric model in place, and patients are not referred to geriatrics or oncogeriatrics. Consequently, they do not participate in multidisciplinary oncology meeting (MDT) or the treatment decision-making process. They are also not involved in perioperative geriatric oncology care. Both highlighted a lack of awareness and interest among other medical specialists, as well as poor communication between healthcare professionals, as limitations. They consider training to be essential for the implementation of oncogeriatrics and would be interested in attending a masterclass on perioperative geriatric medicine.

Austria

*n* = 3 (median survey progress: 100%)

One was a geriatrician (33%), while the other two were internal medicine physicians specialising in geriatrics (67%). They predominantly worked in dedicated geriatric hospitals (67%).

In terms of education, Austria demonstrated a heterogeneous approach: one respondent reported compulsory educational days, another a voluntary educational programme for geriatricians in training, and the third explicitly stated that they received no oncogeriatric education or training. There are no specific guidelines for treating older patients with cancer. In terms of organisational models, two respondents reported no formal oncogeriatric model or referral to geriatrics/oncogeriatrics (67%), whereas the geriatrician reported a two-step model (33%). Referrals, when considered, were age-based in all cases (100%). Referrals were reported to occur mainly in cases of refractory/recurrent disease or when complications/toxicity arise (33% each).

Only the geriatrician reported routinely participating in non-tumour-specific MDT and in the treatment decision-making process. R also described being involved in perioperative geriatric medicine via a liaison model, primarily for hepatobiliary cancer.

Limitations were centred on policy-maker awareness (67%). Suggested solutions pointed towards regulatory measures and education (each supported by at least one respondent). Interest in perioperative geriatric oncology was mixed and incomplete.

Belarus

*n* = 1 (median survey progress: 100%)

The respondent was a geriatrician who works in an outpatient-only setting. R indicated having access to a voluntary educational programme for training geriatricians, and reported the existence of guidelines for certain types of cancer.

Despite this, R reported no formal oncogeriatric model, and patients are not referred to geriatrics/oncogeriatrics. If referral is considered, it is purely age-based at the start of oncological treatment. CGA is performed by non-physician specialists, and the results are shared at a multidisciplinary team meeting. R does not participate in perioperative geriatric oncology medicine.

R highlighted limitations, focusing on a lack of education in geriatric oncology and considered education for geriatricians to be the key solution. R expressed an interest in perioperative geriatric oncology medicine.

Bulgaria

*n* = 1 (median survey progress: 31%)

Single respondent, specialized in geriatrics, who works in an outpatient/ambulatory setting (outpatient only). R reported that there is no oncogeriatric education or training system in the country, nor any available guidelines. Regarding the geriatric oncology model, R said that patients are referred based on the referring physician's clinical judgement, and age is the criterion used. The remaining information is either missing or unspecified.

Croatia

*n* = 1 (median survey progress: 100%)

A geriatrician working in a general, multi-speciality hospital. R reported an obligatory educational system with no specific treatment guidelines for older patients with cancer in his country. According to the geriatric oncology model, referrals are made based on clinical judgement, primarily at diagnosis and at the start of treatment. R indicated nursing involvement in CGA performance, with results discussed at multidisciplinary team meetings and by phone. In this way, R participates in casual/incidental MDTs, being part of the treatment decision process in some cases. R describes a structured form of collaboration with kidney and bladder oncological surgery, where the geriatrician is present on the surgical ward to provide advice and/or co-management.

R identifies nearly all major barriers as limitations and proposes training as a solution. R is interested in a perioperative medicine masterclass.

Estonia

*n* = 2 (median survey progress: 74%)

The survey was answered by a geriatrician and an internal medicine specialist with a focus on geriatrics who works in a general, multi-speciality hospital. They reported that there are no formal oncogeriatric training programmes or treatment guidelines for this subgroup of patients. The internist reported that patients are not referred and that there is no MDT participation or perioperative geriatric oncology model. A limitation to its implementation was described as being that 'geriatrics is not included in the Estonian healthcare system'.

R described an oncogeriatric model based on clinical judgement according to age or ECOG performance status, mostly once a decision has been made to provide palliative care. The remaining information is either missing or unspecified.

Finland

*n* = 7 (median survey progress: 100%)

All respondents were geriatricians working in general, multi-specialty hospitals.

Contradictory answers were reported regarding education and guidelines: four respondents stated that no structured education system exists. Similarly, four respondents reported that guidelines do not exist.

Two approaches to the oncogeriatric care model dominated equally: referrals based on clinical judgement (3/7, 43%) and a two-step referral model (3/7, 43%). Positive frailty screening using the G8 questionnaire was the most commonly reported screening approach (6/7, 86%).

Four respondents (57%) reported routine participation in tumour-specific multidisciplinary meetings. Two respondents described involvement in perioperative geriatric oncology (29%), and one of these reported a structured co-management model (14%).

Perceived barriers were mainly system-level, particularly relating to funding, staffing and awareness. Preferred solutions emphasised training for geriatricians and oncologists.

Finally, six respondents (86%) expressed interest in a perioperative medicine masterclass.

Germany

*n* = 9 (median survey progress: 100%)

The majority were geriatricians (89%), working in both general multi-specialty hospitals (44%) and dedicated geriatric hospitals (56%).

Eight respondents reported having no structured education. Respondents answers regarding guidelines were heterogeneous.

The predominant oncogeriatric care model described was referral-based, relying on the clinical judgement of oncologists and haematologists (67%), mostly for late-stage or needs-based referrals. A sizeable minority reported no referral model (patients were not referred) (33%).

Along these lines, five respondents stated that no geriatric team member participated in the MDT (56%), while three reported incidental participation (33%), and in some cases, participation in the treatment decision process. None of them were involved in managing patients during hospitalisation for oncological surgery, nor did they have a structured form of collaboration.

Regarding perceived barriers, a lack of awareness among other specialists (89%) and policymakers (56%) stood out. The preferred solutions were education for geriatricians and oncologists (both 67%). Seven were interested in a perioperative medicine masterclass.

Greece

*n* = 3 (median survey progress: 100%)

Respondents were mostly internists with a geriatric focus (67%) working mainly in general multi-specialty hospitals (67%). Oncogeriatrics education was largely absent or unknown, as was the existence of specific treatment guidelines. None of the respondents described an oncogeriatric care model; all selected “None, patients are not referred.” They reported that they do not attend MDTs, or do so only incidentally (33%), and they do not collaborate in the perioperative management of oncological patients.

Regarding barriers, all respondents cited a lack of funding, education, communication, and geriatricians. As preferred solutions, there was universal support for enhanced education and training in oncogeriatrics.

Hungary

*n = 1* (median survey progress: 100%)

The respondent was an internist with a geriatric focus working in a general multi-specialty hospital. R reported mandatory educational days for geriatricians and the availability of specific treatment guidelines for older patients with certain cancer types. Regarding the oncogeriatric care model, R described referrals based on clinical judgment within a consultative multidisciplinary approach, in which a multidisciplinary team assesses patients at the start of oncological treatment. R stated that the CGA is performed by a geriatrician in training and/or a specialized nurse practitioner, and that the resulting recommendations are shared during multidisciplinary meetings. R routinely participates in tumour-specific MDTs and is consulted for advice and/or co-management of hospitalized patients undergoing oncological surgery.

As limitations to implementation, R reported lack of time, support staff, funding/reimbursement, and geriatricians. As potential solutions, R emphasized the need for education for both geriatricians and oncologists, and expressed interest in a perioperative medicine masterclass.

Iceland

*n* = 4 (median survey progress: 100%)

All respondents were geriatricians working in general multi-specialty hospitals. There is no structured education or training in geriatric oncology for geriatricians, and no specific treatment guidelines for this subgroup of patients. Regarding the oncogeriatric model of care, 75% reported “None. Patients are not referred,” while the remaining 25% reported referrals based on clinical judgment. In those cases, a geriatrician in training or a nurse practitioner performs the CGA, and the recommendations are shared via a written summary. Any member of the geriatric team routinely participates in MDTs, and none is involved in perioperative geriatric oncology care.

As limitations, 75% perceived a lack of interest/awareness among policy makers and 75% reported a shortage of geriatricians. As solutions, 75% supported advocating for political or stakeholder influence. One respondent was interested in a perioperative medicine masterclass.

Israel

*n* = 3 (median survey progress: 100%)

The three respondents were geriatricians working in general multi-specialty hospitals. Their answers were heterogeneous regarding education and training in geriatric oncology for geriatricians (67% reported none, while 33% reported mandatory training days) and regarding guidelines (ranging from entity-specific cancer guidelines to general clinical recommendations, to none). All three described an oncogeriatric care model in which referrals are based on clinical judgment, either at diagnosis or at the start of treatment. One respondent routinely participates in tumour-specific MDTs, while another attends MDTs only incidentally; one reported being involved in the co-management of surgical oncology patients.

The main perceived barriers were a lack of interest/awareness among other specialists and a shortage of geriatricians. Education for geriatricians and oncologists was proposed as a solution. 67% were interested in a perioperative medicine masterclass.

Lithuania

*n* = 2 (median survey progress: 63%)

Both respondents were geriatricians working in general multi-specialty hospitals. Regarding education and training in geriatric oncology for geriatricians, they reported having voluntary training days but no specific treatment guidelines for older patients with cancer. One respondent reported not having an oncogeriatric model of care, while the other stated that patients are referred based on clinical judgment at diagnosis. However, this respondent also specified not participating in MDTs and not being involved in the care of surgical oncology patients during hospitalization, although R expressed interest in a perioperative medicine masterclass.

Luxembourg

*n* = 1 (median survey progress: 100%).

The respondent works as a geriatrician in a general hospital. R reported having no education or formal training in geriatric oncology for geriatricians and no specific guidelines, only clinical recommendations to treat these patients. Regarding the oncogeriatric care model, R selected referral based on clinical judgment and a two-step model in which a CGA is performed after a positive frailty screening using the G8, at the start of oncological treatment. R stated that to participate only casually (incidentally) in MDTs, without being involved in treatment decision-making. R also reported not being involved in the management of surgical oncology patients during hospitalization.

As barriers, R cited lack of interest among other specialists, lack of time, and lack of education. As proposed solutions, R emphasized education for geriatricians, dual-training/dedicated programs, and regulation (mandatory CGA). R expressed interest in a perioperative medicine masterclass.

Malta

*n* = 3 (median survey progress: 100%)

All respondents were geriatricians working in dedicated geriatric hospitals. They reported no education or formal training in geriatric oncology for geriatricians and no specific treatment guidelines. Regarding the oncogeriatric care model, all three described referrals based on clinical judgment, and one also selected a two-step model. They stated that they do not participate in MDTs and are not involved in the management of hospitalized surgical oncology patients.

The dominant barriers reported were a shortage of geriatricians (67%) and a lack of interest/awareness among other specialists. As solutions, they mainly proposed education and expressed interest in a perioperative medicine masterclass.

Principio del formulario

Final del formulario

Norway

*n* = 7 (median survey progress: 81%)

All respondents were geriatricians working in general multi-specialty hospitals. Formal education in geriatric oncology was limited: 57% reported no geriatric oncology (GO) education, while 29% described voluntary training days and 14% obligatory training days. Respondents answers regarding treatment guidelines were heterogeneous: 29% reported having specific guidelines, whereas 43% reported not having any.

Oncogeriatric care was active and mostly structured. 71% reported referrals based on clinical judgment, and 71% reported using a two-step model. Frailty screening relied heavily on the Clinical Frailty Scale (CFS; 86%) and was mainly performed at the start of treatment (57%).

Geriatric participation in MDTs was largely absent (71% reported no participation of the geriatric team), and they were not involved in the perioperative management of hospitalized surgical oncology patients.

Reported barriers were dispersed, with no single dominant issue; lack of time, lack of funding/reimbursement, low policy-maker awareness, and shortage of geriatricians each appeared in 43% of responses. Proposed solutions leaned toward mandatory CGA/regulatory measures (57%) and education for geriatricians and oncologists (each 43%).

Poland

*n* = 8 (median survey progress 78%)

Respondents were more heterogeneous: 63% were geriatricians, 50% were internists with a geriatrics focus, and 13% reported another profession (categories overlap). Most worked in general multi-specialty hospitals (75%), with a small representation from dedicated geriatric hospitals and specialty cancer centres (12.5% each). Education in geriatric oncology (GO) was mixed: 50% reported none, while smaller proportions reported obliged training days (25%) or voluntary/obliged programs (~12.5% each). They reported no specific treatment guidelines available.

Care models were inconsistent: 50% reported no oncogeriatric referral pathway, 38% relied on clinical judgment, and only 13% used a two-step model. Screening relied predominantly on VES-13 (50%), and referrals generally occurred at later stages or when complications/toxicity arose.

Geriatric participation in MDTs was mostly absent (75%). Only 25% reported involvement in the perioperative management of hospitalized surgical oncology patients.

Reported barriers were mainly lack of interest/awareness among other specialists and policy makers (50% each), and a shortage of geriatricians (50%). Proposed solutions were diverse, led by education for oncologists (50%) and dual-training and regulatory measures (38% each). Overall interest in perioperative medicine was very low.

Romania

*n* = 5 (median survey progress: 100%)

All respondents were geriatricians, and most worked in general multi-specialty hospitals (80%). Formal education in geriatric oncology was absent. Respondents answers regarding guidelines were heterogeneous, although most reported having none.

The geriatric oncology care model was mainly clinician-driven: 80% reported referrals based on clinical judgment, while 20% reported no referral to geriatrics. Geriatric participation in MDTs was completely absent (100% reporting no geriatric team participation). One respondent reported involvement in the perioperative management of hospitalized surgical oncology patients through a liaison model for bladder, kidney, and prostate cancer.

Perceived barriers were highly consistent: lack of education in geriatric oncology was universal (100%), accompanied by low awareness/interest among policy makers (80%) and among other specialists (60%). Proposed solutions showed strong system-level alignment: 100% supported education for both geriatricians and oncologists/haematologists/radiotherapists, implementation of dual-training or dedicated geriatric oncology pathways, and engagement with political/European stakeholders.

Serbia

*n* = 1 (median survey progress 100%)

The respondent was a geriatrician working in a general multi-specialty hospital. No structured GO education existed, and the respondent was unaware of any guidelines. In practice, oncogeriatric care was not implemented: patients were not referred to geriatrics. When referral occurred, it happened only at the palliative decision stage. Geriatric participation in MDTs was absent. However, R did take part in the management of hospitalized surgical oncology patients, mainly for gastrointestinal cancers, without any formal or structured collaboration. Barriers were framed primarily as awareness/interest gaps, lack of education, communication problems, and a shortage of geriatricians, while proposed solutions emphasized education for geriatrics and oncology, political/advocacy engagement, and regulatory measures. The respondent also expressed interest in a perioperative medicine masterclass.

Slovakia

*n* = 1 (median survey progress 100%)

This respondent identified as both a geriatrician and an internist with a geriatric focus, and was based in a general multi-specialty hospital. Formal GO education was absent, as were specific treatment guidelines. Oncogeriatric referral existed only informally, driven by clinical judgment and age rather than frailty screening. Geriatric MDT involvement was limited to casual/incidental meetings rather than structured tumour boards. Respondent reported being involved, through a liaison model, in the management of hospitalized surgical oncology patients and expressed interest in a masterclass. The main obstacles highlighted were low policy prioritization and lack of time, and the preferred solutions were targeted education for geriatricians (and for surgeons on the surgical side).

Slovenia

*n* = 1 (median survey progress 100%)

The respondent was an internist with a geriatric focus (not categorized as a geriatrician), working in a general multi-specialty hospital. No GO training programs or specific guidelines were available. Despite selecting frailty screening as the theoretical referral trigger, the reported care model was “none—patients are not referred,” indicating a disconnect between screening intent and actual referral pathways. Screening relied exclusively on VES-13, with no other tools used, and CGA was performed by unspecified “other” staff. Advice was generated and documented in the patient file but not discussed in MDTs. Geriatrics did not participate in MDTs or in the management of hospitalized surgical oncology patients. Barriers focused on workforce and engagement issues, and proposed solutions leaned toward education for geriatrics and oncology plus policy/regulatory actions, but not dual-training programs. The respondent expressed interest in a perioperative medicine masterclass.

Sweden

*n* = 2 (median survey progress: 100%)

Both respondents were geriatricians, though one also identified with internal medicine with a geriatrics focus, and the other specified palliative medicine. Both worked in general multi-specialty hospitals. No specific educational formats were available, and responses were split between “no education” and “don’t know”; they also reported no specific guidelines. Care delivery was heterogeneous: one centre reported no referral model, while the other used a two-step screen-then-refer approach. Geriatrics did not participate in MDTs in either setting. One respondent reported a liaison model for managing hospitalized surgical oncology patients. The dominant barrier was a lack of policy-maker awareness (100%), with additional gaps in the workforce and education. Proposed solutions showed strong consensus for a system-level package: education for geriatricians and oncologists, dual-training programs, political advocacy, and regulatory/mandatory CGA measures.

Switzerland

*n* = 3 (median survey progress: 100%)

All respondents were geriatricians working in general multi-specialty hospitals. Geriatric oncology (GO) education was limited and patchy: one reported voluntary training days, one an obligatory program, and one no training at all, with all other education formats absent. Guideline availability and use were unclear, given the heterogeneous responses. The care model was maximally heterogeneous, with each respondent describing a different system (ranging from clinical-judgement referral to a two-step pathway and a multidisciplinary consultative model). Advice was routinely generated, and results were commonly integrated into MDTs (two-thirds), which is a notable marker of service maturity. Referrals could occur early and throughout the care pathway, but follow-up remained selective rather than routine. Regarding perioperative medicine, one respondent described a liaison model for managing hospitalized prostate cancer patients. Barriers centred on engagement from other specialists, lack of GO education, and a shortage of geriatricians (each reported by about two-thirds). Proposed solutions were less consensual: most system-level measures received only minority support, except for universal endorsement of education for surgical specialists.
