# Supplementary material for: A survey among geriatricians on geriatric oncology and geriatric surgical oncology models in Europe
Source: Eur Geriatr Med. 2026 Aug 28;17(5):3017–36. doi: 10.1007/s41999-026-01575-y (PMC13755084; doi:10.1007/s41999-026-01575-y)
Supplement: Supplementary file 5 [file 41999_2026_1575_MOESM5_ESM.docx]

**Supplementary Table S1. Barriers and improvements for geriatric oncology in your institution**

|  | All countries^1^ n= 395 (%) | Italy *n*=66 (%) | France *n*= 47 (%) | Spain *n*= 47 (%) | Netherlands *n*= 35 (%) | Denmark *n*= 32 (%) | United Kingdom *n*= 31 (%) | Turkey *n*= 26 (%) | Ireland *n*= 14 (%) | Portugal *n*= 13 (%) | Belgium *n*= 12 (%) |
| --- | --- | --- | --- | --- | --- | --- | --- | --- | --- | --- | --- |
| **What do you think are the main limitations for the development of medical geriatric oncology in your country?*** | | | | | | | | | | | |
| Lack of awareness and interest among geriatricians | 54 (13.7) | 16 (24.2) | 0 (0.0) | 5 (10.6) | 0 (0.0) | 4 (12.5) | 8 (25.8) | 6 (23.1) | 2 (14.3) | 2 (15.4) | 0 (0.0) |
| Lack of awareness and interest among other medical specialists (e.g. oncologist, surgeons) | 187 (47.3) | 29 (43.9) | 21 (44.7) | 19 (40.4) | 23 (65.7) | 7 (21.9) | 16 (51.6) | 18 (69.2) | 3 (21.4) | 7 (53.8) | 7 (58.3) |
| Lack of awareness and interest among policy makers | 137 (34.7) | 22 (33.3) | 17 (36.2) | 17 (36.2) | 6 (17.1) | 6 (18.8) | 14 (45.2) | 10 (38.5) | 3 (21.4) | 5 (38.5) | 5 (41.7) |
| Lack of evidence | 28 (7.1) | 6 (9.1) | 3 (6.4) | 1 (2.1) | 7 (20.0) | 2 (6.2) | 3 (9.7) | 0 (0.0) | 0 (0.0) | 1 (7.7) | 1 (8.3) |
| Lack of time | 131 (33.2) | 15 (22.7) | 25 (53.2) | 19 (40.4) | 19 (54.3) | 5 (15.6) | 13 (41.9) | 8 (30.8) | 1 (7.1) | 3 (23.1) | 4 (33.3) |
| Lack of support staff | 124 (31.4) | 12 (18.2) | 20 (42.6) | 18 (38.3) | 13 (37.1) | 4 (12.5) | 15 (48.4) | 9 (34.6) | 2 (14.3) | 5 (38.5) | 5 (41.7) |
| Lack of funding/reimbursement | 118 (29.9) | 12 (18.2) | 15 (31.9) | 11 (23.4) | 8 (22.9) | 5 (15.6) | 20 (64.5) | 5 (19.2) | 2 (14.3) | 6 (46.2) | 8 (66.7) |
| Lack of education on geriatric oncology | 167 (42.3) | 31 (47.0) | 16 (34.0) | 17 (36.2) | 13 (37.1) | 8 (25.0) | 15 (48.4) | 18 (69.2) | 5 (35.7) | 9 (69.2) | 4 (33.3) |
| Lack of communication between involved healthcare professionals | 107 (27.1) | 21 (31.8) | 14 (29.8) | 11 (23.4) | 8 (22.9) | 4 (12.5) | 6 (19.4) | 13 (50.0) | 1 (7.1) | 3 (23.1) | 2 (16.7) |
| Lack of geriatricians | 175 (44.3) | 18 (27.3) | 35 (74.5) | 19 (40.4) | 7 (20.0) | 12 (37.5) | 18 (58.1) | 13 (50.0) | 2 (14.3) | 10 (76.9) | 7 (58.3) |
| Other^2^ | 8 (2.0) | 1 (1.5) | 1 (2.1) | 0 (0.0) | 2 (5.7) | 0 (0.0) | 0 (0.0) | 0 (0.0) | 0 (0.0) | 0 (0.0) | 0 (0.0) |
| **What do you think are the main limitations for the development of surgical geriatric oncology in your country?*** | | | | | | | | | | | |
| Lack of awareness and interest among geriatricians | 50 (12.7) | 15 (22.7) | 1 (2.1) | 4 (8.5) | 1 (2.9) | 2 (6.3) | 5 (16.1) | 7 (26.9) | 1 (7.1) | 3 (23.1) | 0 (0.0) |
| Lack of awareness and interest among other medical specialists (e.g. oncologist, surgeons) | 175 (44.3) | 25 (37.9) | 25 (53.2) | 23 (48.9) | 17 (48.6) | 6 (18.8) | 10 (32.3) | 18 (69.2) | 2 (14.3) | 6 (46.2) | 6 (50.0) |
| Lack of awareness and interest among policy makers | 130 (32.9) | 18 (27.3) | 13 (27.7) | 15 (31.9) | 6 (17.1) | 8 (25.0) | 10 (32.3) | 9 (34.6) | 4 (28.6) | 5 (38.5) | 7 (58.3) |
| Lack of evidence | 35 (8.9) | 8 (12.1) | 5 (10.6) | 1 (2.1) | 8 (22.9) | 4 (12.5) | 1 (3.2) | 2 (7.7) | 0 (0.0) | 1 (7.7) | 0 (0.0) |
| Lack of time | 129 (32.7) | 15 (22.7) | 25 (53.2) | 18 (38.3) | 15 (42.9) | 2 (6.3) | 14 (45.2) | 11 (42.3) | 2 (14.3) | 3 (23.1) | 4 (33.3) |
| Lack of support staff | 119 (30.1) | 11 (16.7) | 20 (42.6) | 14 (29.8) | 12 (34.3) | 3 (9.4) | 16 (51.6) | 9 (34.6) | 2 (14.3) | 5 (38.5) | 3 (25.0) |
| Lack of funding/reimbursement | 103 (26.1) | 8 (12.1) | 12 (25.5) | 10 (21.3) | 9 (25.7) | 3 (9.4) | 16 (51.6) | 6 (23.1) | 2 (14.3) | 5 (38.5) | 6 (50.0) |
| Lack of education on geriatric oncology | 147 (37.2) | 29 (43.9) | 13 (27.7) | 16 (34.0) | 10 (28.6) | 9 (28.1) | 9 (29.0) | 17 (65.4) | 3 (21.4) | 8 (61.5) | 2 (16.7) |
| Lack of communication between involved healthcare professionals | 119 (30.1) | 22 (33.3) | 13 (27.7) | 15 (31.9) | 8 (22.9) | 4 (12.5) | 8 (25.8) | 17 (65.4) | 1 (7.1) | 3 (23.1) | 1 (8.3) |
| Lack of geriatricians | 167 (42.3) | 16 (24.2) | 34 (72.3) | 18 (38.3) | 6 (17.1) | 12 (37.5) | 16 (51.6) | 14 (53.8) | 2 (14.3) | 9 (69.2) | 6 (50.0) |
| Other^3^ | 5 (1.3) | 1 (1.5) | 2 (4.3) | 0 (0.0) | 1 (2.9) | 0 (0.0) | 0 (0.0) | 0 (0.0) | 0 (0.0) | 0 (0.0) | 0 (0.0) |
| **What solutions or actions do you envision to promote the development of geriatric medical oncology (oncology, hematology, radiotherapy) in your country?*** | | | | | | | | | | | |
| Education for geriatricians | 189 (47.8) | 33 (50.0) | 22 (46.8) | 21 (44.7) | 15 (42.9) | 9 (28.1) | 14 (45.2) | 16 (61.5) | 5 (35.7) | 8 (61.5) | 5 (41.7) |
| Education for oncologists, haematologists, radiotherapists | 277 (57.5) | 32 (48.5) | 29 (61.7) | 30 (63.8) | 22 (62.9) | 6 (18.8) | 21 (67.7) | 19 (73.1) | 5 (35.7) | 12 (92.3) | 8 (66.7) |
| Implementation of dual-training programs or dedicated geriatric oncology fellowships | 190 (48.1) | 32 (48.5) | 22 (46.8) | 31 (66.0) | 17 (48.6) | 8 (25.0) | 13 (41.9) | 16 (61.5) | 5 (35.7) | 11 (84.6) | 5 (41.7) |
| Influence on political stake-holders (e.g. by European medical societies) | 164 (41.5) | 29 (43.9) | 14 (29.8) | 22 (46.8) | 11 (31.4) | 9 (28.1) | 19 (61.3) | 9 (34.6) | 5 (35.7) | 10 (76.9) | 3 (25.0) |
| Regulatory solutions (e.g. obligatory use of (C)GA at certified cancer centres) | 167 (42.3) | 23 (34.8) | 27 (57.4) | 22 (46.8) | 8 (22.9) | 4 (12.5) | 15 (48.4) | 15 (57.7) | 4 (28.6) | 10 (76.9) | 8 (66.7) |
| Other^4^ | 14 (3.5) | 1 (1.5) | 2 (4.3) | 0 (0.0) | 2 (5.7) | 3 (9.4) | 0 (0.0) | 1 (3.8) | 1 (7.1) | 2 (15.4) | 0 (0.0) |
| **What solutions or actions do you envision to promote the development of surgical geriatric oncology in your country?*** | | | | | | | | | | | |
| Education for geriatricians | 174 (44.1) | 33 (50.0) | 21 (44.7) | 18 (38.3) | 14 (40.0) | 9 (28.1) | 10 (32.3) | 17 (65.4) | 4 (28.6) | 8 (61.5) | 4 (33.3) |
| Education for surgical specialists | 232 (58.7) | 34 (51.5) | 34 (72.3) | 33 (70.2) | 25 (71.4) | 5 (15.6) | 20 (64.5) | 21 (80.8) | 4 (28.6) | 11 (84.6) | 5 (41.7) |
| Implementation of dual-training programs or dedicated geriatric oncology fellowships | 169 (42.8) | 29 (43.9) | 17 (36.2) | 28 (59.6) | 16 (45.7) | 7 (21.9) | 10 (32.3) | 14 (53.8) | 3 (21.4) | 10 (76.9) | 6 (50.0) |
| Influence on political stake-holders (e.g. by European medical societies) | 151 (38.2) | 28 (42.4) | 16 (34.0) | 22 (46.8) | 10 (28.6) | 7 (21.9) | 15 (48.4) | 8 (30.8) | 3 (21.4) | 11 (84.6) | 3 (25.0) |
| Regulatory solutions (e.g. obligatory use of (C)GA at certified cancer centres) | 165 (41.8) | 25 (37.9) | 22 (46.8) | 22 (46.8) | 10 (28.6) | 2 (6.3) | 16 (51.6) | 15 (57.7) | 3 (21.4) | 9 (69.2) | 8 (66.7) |
| Other^5^ | 11 (2.8) | 1 (1.5) | 2 (4.3) | 0 (0.0) | 2 (5.7) | 2 (6.3) | 0 (0.0) | 0 (0.0) | 1 (7.1) | 1 (7.7) | 0 (0.0) |
| **Would you be interested in a masterclass on perioperative geriatric medicine?^6^** | | | | | | | | | | | |
| Yes | 250 (80.6) | 35 (71.4) | 35 (81.4) | 37 (90.2) | 21 (75.0) | 11 (78.6) | 19 (73.1) | 20 (87.0) | 5 (100.0) | 12 (100.0) | 9 (81.8) |
| No | 25 (8.1) | 6 (12.2) | 4 (9.3) | 1 (2.4) | 2 (7.1) | 1 (7.1) | 6 (23.1) | 0 (0.0) | 0 (0.0) | 0 (0.0) | 0 (0.0) |
| I don’t know | 35 (11.3) | 8 (16.3) | 4 (9.3) | 3 (7.3) | 5 (17.9) | 2 (14.3) | 1 (3.8) | 3 (13.0) | 0 (0.0) | 0 (0.0) | 2 (18.2) |

* Multiple response questions (where respondents can select one or more options)

^1^All countries included all respondents, geriatricians and/or internists with a focus on geriatrics, with ≥ 26% progression.

^2^Challenges include ageism, differing expectations, lack of patient awareness, the absence of multidisciplinary wards, and a shortage of nurse practitioners. A respondent from Estonia noted that geriatrics is not integrated into the healthcare system.

^3^Ageism, differing expectations, and a shortage of liaison nurses.

^4^Research initiatives, remote support systems, and clinical guidelines for managing cancer in older adults

^5^Training for anesthesiologists, guidelines for older patients with cancer, more staff to carry out the CGA, study projects.

^6^Percentages exclude missing values. Missing by country (not included in %): All *n*= 85, Italy *n*=17, France *n*=4, Spain *n*=6, Netherlands *n*=7, Denmark *n*=18, United Kingdom *n*=5, Turkey *n*=3, Ireland *n*=9, Portugal *n*=1, Belgium *n*=1.
